# Planar cell polarity emerges through polarized accumulation of Wnt11

**DOI:** 10.1126/sciadv.aea0326

**Published:** 2026-02-25

**Authors:** Yusuke Mii, Minako Suzuki, Hiroshi Koyama, Kei Nakayama, Ritsuko Takada, Tomoe Kobayashi, Motosuke Tsutsumi, Tomomi Nemoto, Makoto Matsuyama, Toshihiko Fujimori, Shinji Takada

**Affiliations:** ^1^ National Institute for Basic Biology, National Institutes of Natural Sciences, 5-1 Higashiyama, Myodaiji-cho, Okazaki, Aichi 444-8787, Japan.; ^2^ Exploratory Research Center on Life and Living Systems (ExCELLS), National Institutes of Natural Sciences, 5-1 Higashiyama, Myodaiji-cho, Okazaki, Aichi 444-8787, Japan.; ^3^ The Graduate University for Advanced Studies (SOKENDAI), 5-1 Higashiyama, Myodaiji-cho, Okazaki, Aichi 444-8787, Japan.; ^4^ Institute for Life and Medical Sciences, Kyoto University, 53 Shogoin Kawahara-cho, Sakyo-ku, Kyoto 606-8507, Japan.; ^5^ Department of Polymer Chemistry, Graduate School of Engineering, Kyoto University, Kyoto 606-8507, Japan.; ^6^ PRESTO, Japan Science and Technology Agency (JST), Kawaguchi, Saitama 332-0012, Japan.; ^7^ Kobe Pharmaceutical University, 4-19-1 Motoyamakitamachi, Higashinada-ku, Kobe 658-8558, Japan.; ^8^ Shigei Medical Research Institutes, 2117 Yamada, Minami-ku, Okayama 701-0202, Japan.; ^9^ National Institute for Physiological Sciences, National Institutes of Natural Sciences, 5-1 Higashiyama, Myodaiji-cho, Okazaki, Aichi 444-8787, Japan.

## Abstract

Planar cell polarity (PCP) is established by asymmetric localization of core PCP components in each cell. Because some Wnt proteins can induce PCP in vertebrates, it has been proposed that Wnt concentration gradients provide spatial cues for polarization. However, here, we present evidence that Wnt11 can regulate PCP in a gradient-independent manner. In the neural plate of *Xenopus* embryos, endogenous Wnt11 does not form an obvious gradient in the direction of PCP, but is polarized at cell boundaries together with core PCP components. Wnt11 polarization is dependent on core PCP components, while polarization of core PCP components can also be induced by Wnt11, indicating a mutual amplification loop between Wnt11 and core PCP components in PCP formation. Furthermore, Wnt11 and core PCP components compose an intercellular loop to coordinate the direction of polarity. Thus, we propose that local and reciprocal interactions between Wnt11 and core PCP components can generate PCP.

## Teaser

Polarized but not graded Wnt11 underlies emergence of planar cell polarity.

## INTRODUCTION

Diffusible signals regulate many biological phenomena, from directional control of migration in single-cell organisms to development and homeostasis of multicellular tissues. In these cases, diffusible signals are thought to act in a top-down manner. For instance, in patterning of developing tissues, it has been thought that these molecules form a concentration gradient around the producing cells, and that positional information provided by the concentration gradient is sensed by receiving cells, thereby orchestrating tissue development (*1*–*3*). However, it remains unclear whether diffusible signals always exert top-down control over recipient cells, or whether formation of a concentration gradient is actually important in the action of diffusible signals.

Planar cell polarity (PCP) is the collective alignment of individual cell polarities in a tissue plane (*4*–*10*). A classical example of PCP is observed in *Drosophila* wing, in which the hair of each cell is aligned in coordinated fashion toward the distal side (*4*, *11*). In vertebrates, the alignment of stereocilia bundles in hair cells in the inner ear (*12*) and the orientation of hair follicles in the skin (*13*–*15*) are examples of PCP. Proteins essential for the establishment of PCP are called core PCP components. These include three cell membrane proteins, Frizzled (Fzd) (*16*), Van Gogh-like (Vangl) (*17*, *18*), and Celsr [or Framingo (Fmi)] (*19*), and two intracellular proteins, Disheveled (Dvl or Dsh in *Drosophila*) (*20*, *21*) and Prickle (Pk) (*22*), which interact with Fzd and Vangl, respectively. Fzd and Dvl as well as Vangl and Pk are localized in an asymmetric manner at interfaces of adjacent cells, resulting in polarization of cell structures. One of the mysteries of PCP formation is how individual cell polarities are aligned. One possible answer to this question is that a “global cue” controls the polarity of each cell. Because PCP can be directed by locally expressed Wnt in *Drosophila* and frog embryos (*23*, *24*), a model has been proposed in which the polarity direction of each cell is determined by the direction of a Wnt concentration gradient (*7*). Consistently, some mutants of Wnt genes, as well as loss of function of components of a noncanonical Wnt signaling pathway, the Wnt/PCP pathway, exhibit abnormalities in PCP or PCP-related phenotypes including convergent extension movements in vertebrates (*25*–*28*), although it is still debatable whether Wnt ligands are required for PCP formation in *Drosophila* (*23*, *29*–*31*). In the “global cue model”, Wnt ligands secreted from producing cells form a concentration gradient, along which each cell establishes polarity (*4*, *7*). If we assume that this gradient covers the entire tissue in which PCP is formed, the gradient must be shallow, because such a tissue is usually much larger than the area of signal-producing cells. However, in this situation, for each cell to sense the direction of the concentration gradient, cells must be sensitive enough to read very slight concentration differences. On the other hand, if we assume that the sensitivity of the cells is high enough, the gradient must be very smooth and precise; otherwise, the direction of PCP will be disturbed. It remains unclear how Wnt is distributed in tissues where PCP is formed, but the smoothness of Wnt signaling gradient is disturbed by noise during morphogenesis along the anterior-posterior (A-P) axis in zebrafish embryos (*32*). Therefore, although Wnt is actually required for PCP formation in many vertebrate tissues, it remains unclear whether the “global cue model” is adequate to explain the function of Wnt in PCP formation.

It has been shown in *Drosophila* and mice that interactions between neighboring cells are important for PCP formation (*33*–*35*). These lines of evidence imply the importance of local cell-to-cell interaction for PCP formation. For instance, mouse chimeras consisting of PCP-sufficient cells and cells that cannot form PCP indicate that core PCP components cannot be polarized in a cell-autonomous manner. Rather, PCP-mediated contacts along single cell-cell interfaces are sufficient to polarize core PCP components (*35*). These results show that intercellular interactions among core PCP components are critical for PCP formation. However, the molecular mechanism underlying these intercellular interactions and the relationship between them and Wnt signal remains unclear.

To better understand the function of Wnt signaling in PCP formation, it is important to examine the spatial distribution of Wnt. Wnt distribution in embryos has been studied in invertebrates and vertebrates. The most well-known example is a series of analyses of the spatial distribution of Wingless (Wg, product of *Wnt1*-ortholog) in *Drosophila* (*36*–*39*). In addition, distributions of endogenous Wnt ligands in vertebrate embryos have been reported (*40*, *41*). Some of these studies show that Wg/Wnt was localized on cell surfaces in punctate patterns (*36*, *38*, *41*), suggesting that it accumulates locally on cell surfaces (*42*). However, the relationship between localization of Wnt proteins and PCP has been poorly understood. In this study, to observe Wnt distribution in PCP formation, we created a Wnt11 antibody that can be used for immunostaining of embryos, and we analyzed the distribution of endogenous Wnt11 using *Xenopus* embryos. In contrast to prevailing models, in the neural plate, where PCP is established, Wnt11 was not distributed in a graded manner, but was polarized at cell boundaries, similar to core PCP components. Unexpectedly, even if Wnt is expressed ubiquitously, i.e., without any direction, core PCP components polarize autonomously, and, at the same time, Wnt11 also polarizes, depending on core PCP components. This polarization can be generated by a positive amplification loop between Wnt11, Fzd7, and phosphorylated Vangl2 in a cis-configuration, and it can be transmitted to adjacent cells via asymmetric assembly of PCP components at cell boundaries. Thus, although Wnt11 and core PCP components do not initially have spatially biased distributions, their interactions and assembly spontaneously generate polarity in cells and tissues. On the basis of these results, we propose that Wnt11 does not regulate PCP in a top-down manner as a global cue, but rather can generate tissue polarity in a bottom-up manner by assembling locally with other proteins in cell membranes.

## RESULTS

### Wnt11 is polarized in PCP formation

**Fig. 1.**
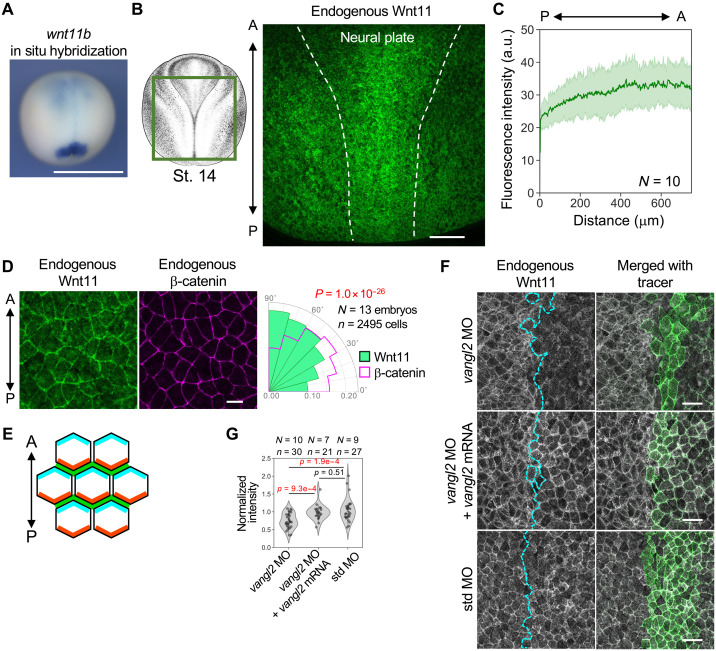
Wnt11 exhibits polarized localization in the neural plate in a PCP-dependent manner. (**A**) Expression pattern of *wnt11* transcripts in early neurula (St. 13). Posterior-dorsal view. Expression of *wnt11b.L* mRNA was visualized by whole-mount in situ hybridization with an antisense probe. (**B**) Immunostaining of endogenous Wnt11 in *Xenopus* embryos (St. 14). The observed area was as illustrated (left). The neural plate region is outlined with dashed lines. The anterior-posterior (A-P) axis is indicated. (**C**) Quantification of Wnt11 along the midline in (B). The light green area indicates one standard deviation. (**D**) Polarized localization of Wnt11. A magnified image of the middle region of Wnt11 immunostaining is presented. β-Catenin was also stained as a membrane marker without polarized localization. Polarity angles of Wnt11 (green) and β-catenin (magenta) are presented with the rose diagram (histogram). Wnt11 exhibits significantly polarized localization toward 90° (corresponding to horizontal localization), compared to β-catenin. Kuiper two-sample test (a circular analog of the Kolmogorov-Smirnov test) test was used for statistical analysis. (**E**) Schematic illustration of localization of Wnt11 (green) and core PCP components (cyan and orange). (**F**) Knockdown of *vangl2* with *vangl2* MO reduced membrane localization of Wnt11. MOs with or without *vangl2* mRNA and membrane tracer (*mRuby2-krasCT* mRNA) were coinjected into the right dorsal blastomere of four-cell embryos, targeting the future neural plate. Boundaries of tracer-negative and tracer-positive areas are indicated with cyan dashed lines. (**G**) Quantification of results shown in (F). Numbers of embryos (*N*) and numbers of regions of interest (ROIs) (*n*) are as indicated. Numbers of embryos (*N*) and numbers of cells (*n*) are as indicated. Amounts of mRNAs/MOs (in nanograms per embryo): *mRuby2-krasCT*, 0.067; *vangl2* MOs, 14; and std MO, 14. Scale bars, 1 mm (A), 200 μm (B), 20 μm (D), and 50 μm (F). Representative data from two independent experiments are presented. a.u., arbitrary unit. *Xenopus* illustrations © Natalya Zahn (2022) (CC BY-NC, https://creativecommons.org/licenses/by-nc/4.0/deed.en).

Transcripts for *Xenopus* Wnt11 (*wnt11b.L*) are strongly expressed in the posterior embryonic region during late gastrula [Stage (St.) 12] to early neurula (St. 14), which is the critical period for establishment of PCP in the neural plate (Fig. 1A and fig. S1A) (*43*, *44*). In addition, its expression was detectable in the anterior and middle regions of neural plate after St. 13 (Fig. 1A and fig. S1A). To examine whether Wnt11 proteins are distributed in a graded manner from these sources, we performed whole-mount immunostaining using a newly generated anti-*Xenopus* Wnt11 antibody (for specificity, see fig. S1, B and C). Wnt11 staining became intense in the neural plate during St. 13 and 14, but it did not show a graded distribution in the posterior-to-anterior direction (Fig. 1, B and C, and fig. S1, E and F). In earlier stages (St. 9 to 11), Wnt11 staining was weak and mostly found inside cells without showing an obvious gradient (fig. S1D). Unexpectedly, at St. 14, when PCP became apparent, imaging at higher magnification revealed that Wnt11 preferentially accumulated on cell boundaries in the mediolateral direction of embryos (Fig. 1, D and E). This result suggests that while Wnt11 can induce PCP signaling, Wnt11 may also be regulated by PCP. Actually, antisense morpholino oligo (MO)–mediated knockdown of a core PCP component gene, *vangl2*, reduced cell membrane–localized Wnt11 (Fig. 1, F and G). These results suggest that Wnt11 localization and abundance on the cell membrane are under control of PCP in *Xenopus* embryos.

**Fig. 2.**
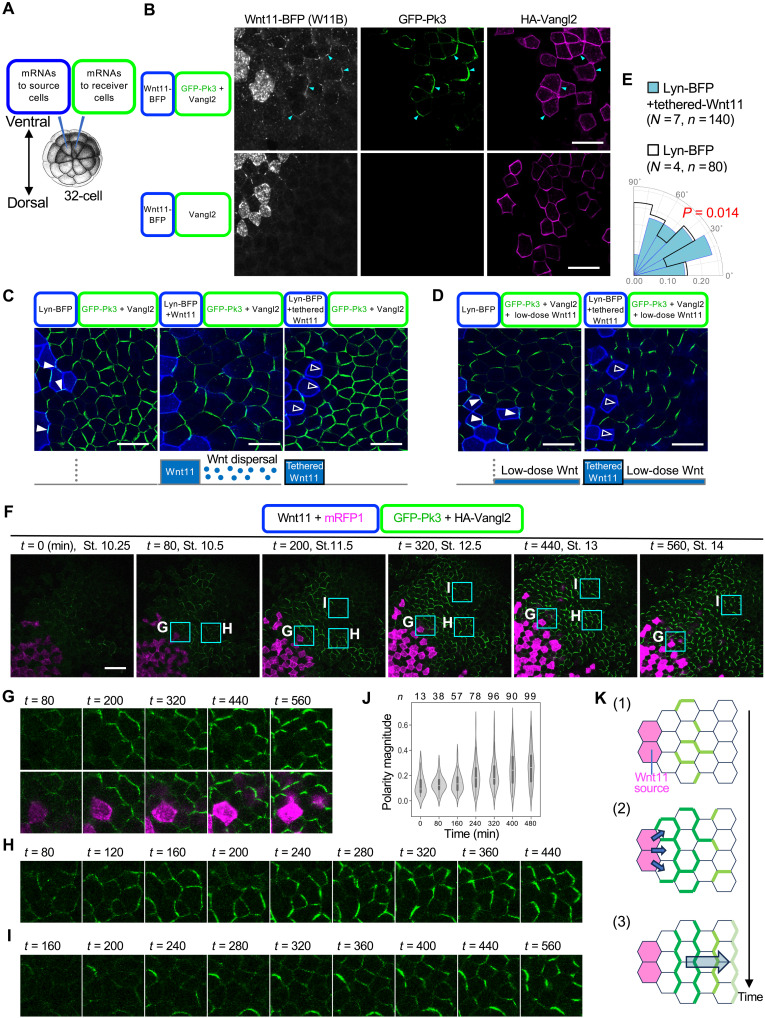
Spatial bias–driven and bias-free roles of Wnt11 for PCP and spatiotemporal profiling of Wnt-dependent polarization of GFP-Pk3. (**A**) Schematic illustration of mRNA injection for rPCP. *Xenopus* embryos injected with mRNAs at the 32-cell stage were observed at St. 14. (**B**) Localization of Wnt11 depends on rPCP. Arrowheads indicate accumulations of Wnt11-BFP with GFP-Pk3 in a region in which GFP-Pk3 and HA-Vangl2 are sparsely expressed. Ectopic HA-Vangl2 without GFP-Pk3 is not sufficient for localization of Wnt11-BFP. (**C**) Tethered-Wnt11 cannot polarize GFP-Pk3 at long range, but does in adjacent cells. Note the presence (closed arrowheads) and absence (open arrowheads) of GFP-Pk3 at boundaries facing tracer- and tethered-Wnt11–expressing cells, respectively. (**D**) Ubiquitously expressed Wnt11 can polarize GFP-Pk3 without direction, and when combined with direction by tethered-Wnt11, polarization can be aligned over long range. Note the presence (closed arrowheads) and absence (open arrowheads) of GFP-Pk3, respectively. (**E**) Quantification of polarity angles with or without direction by tethered-Wnt11. Numbers of embryos (*N*) and numbers of cells (*n*) are as indicated. (**F** to **I**) Time-lapse imaging of rPCP formation in the animal cap region. mRFP1 was used as a tracer for Wnt11-expressing cells. (F) Overall, polarization appears to propagate from the vicinity of the Wnt11 source to distant regions. [(G) to (I)] Subregions adjacent [(G) and (H)] or distant (I) to the source are enlarged. (**J**) Quantification of polarity magnitude of GFP-Pk3 in the time-lapse imaging. Numbers of analyzed cells (*n*) are as indicated. (**K**) Schematic illustrations of PCP formation in rPCP. mRNAs were injected into ventral animal blastomeres at the 32-cell stage, as illustrated. Amounts of mRNAs (in picograms per embryo): *wnt11-BFP*, 500; *lyn-BFP*, 50; *GFP-pk3*, 200 [(B) to (E)] and 100 [(F) to (J)]; *HA-vangl2*, 100 [(B) to (E)] and 50 [(F) to (J)]; *wnt11,* 500 [(C) and (F) to (J)] and 50 (D); *tethered-wnt11*, 500; and *mRFP1*, 500. Scale bars, 50 μm. Representative data from two [(B) to (E)] or three [(F) to (J)] independent experiments are presented. *Xenopus* illustrations © Natalya Zahn (2022) (CC BY-NC, https://creativecommons.org/licenses/by-nc/4.0/deed.en).

In contrast, Wnt11 can induce PCP. For instance, PCP is ectopically established by Wnt11 in a reconstruction system using *Xenopus* embryos (*24*), referred to hereafter as reconstructed PCP (rPCP). In this system, Wnt11 is ectopically expressed in cells (source cells) adjacent to those expressing green fluorescent protein (GFP)–tagged Pk3 (GFP-Pk3) and hemagglutinin (HA)-tagged Vangl2 (HA-Vangl2) (reporter cells) in the animal cap region (Fig. 2A), where PCP is rarely generated (*24*). To visualize Wnt11 in this system, we used mTagBFP2-tagged Wnt11 [Wnt11-BFP (W11B)], which retained the ability to polarize GFP-Pk3 in rPCP (fig. S2A). W11B exhibited polarized colocalization with GFP-Pk3 on reporter cells (Fig. 2B and fig. S2A; this colocalization was apparent in a region in which GFP-Pk3 and HA-Vangl2 are sparsely expressed). Furthermore, W11B polarization was also dependent on PCP because it was not observed in the absence of GFP-Pk3 (Fig. 2B). Thus, while Wnt11 induces PCP, PCP also localizes Wnt11 in rPCP.

### Wnt11 can induce PCP by a combination of its spatial bias–driven and bias-free functions

To detail the reciprocal relationship between Wnt and PCP in rPCP, we focused on how Wnt regulates PCP formation. To examine whether dispersal of Wnt11 is required for polarization, unbound Wnt11 was replaced with membrane-tethered Wnt11-CD8-mCherry (denominated tethered-Wnt11). As predicted, this replacement abolished GFP-Pk3 polarization in reporter cells more than two cells away from cells expressing tethered-Wnt11 (Fig. 2C), indicating that diffusion-mediated dispersal of Wnt11 is required for long-range polarization. On the other hand, cell membrane localization of GFP-Pk3 was specifically abolished from the border adjacent to source cells (Fig. 2C and fig. S2B), suggesting that Wnt11 tethered to cell membranes can polarize neighboring cells. When low-dose Wnt11 was additively and evenly expressed in reporter cells, long-range PCP was generated, directed toward tethered-Wnt11 expressing cells (Fig. 2, D and E). Thus, although Wnt11 dispersal is required for PCP formation, a Wnt11 gradient in reporter cells is likely not essential for PCP formation.

Even in the absence of Wnt11 expression in source cells, additively and evenly expressed Wnt11 polarized GFP-Pk3 in reporter cells, although the direction of cell polarity was not consistent (Fig. 2D). Thus, even when acting without directional bias, uniformly expressed Wnt11 can generate cell polarity. In this case, because Wnt11-expressing cells are not localized in cell populations, the polarity direction is not determined by spatial bias in Wnt11 expression.

On the basis of these results, we speculate that Wnt11 contributes to PCP formation by at least two means. First, Wnt11 can induce polarization of a cell population even in a manner that does not provide directional information, such as a concentration gradient. In rPCP, this function appears to be contributed by Wnt11 molecules that diffuse more than 1 cell diameter from source cells. Second, Wnt11 from source cells can polarize adjacent cells, providing polarity direction toward Wnt-source cells. We refer to these two functions as Wnt bias–free and Wnt bias–driven polarization, respectively. These two functions likely represent characteristics of Wnt11-dependent formation of PCP such that, together, they generate PCP.

### Live-imaging supports two distinct functions of Wnt11 in PCP formation

To see whether these two functions of Wnt11 are actually observed in formation of PCP, we followed dynamic processes in rPCP. By live imaging of this system from St. 10.25 to St. 14, we succeeded in observing the entire duration of PCP establishment (movie S1 and Fig. 2F for cropped images). At the beginning, *t* = 0, GFP-Pk3 was very weakly localized on cell membranes. Around *t* = 80 min, which corresponds to St. 10.5, the first indications of biased localization of GFP-Pk3 were detected in cells some distance from, but not adjacent to, Wnt11 source cells (Fig. 2, G and H). At this time, the direction of GFP-Pk3 polarity was not persistent, fluctuating during a period of 20 to 40 min (Fig. 2H). On the other hand, in cells adjacent to Wnt11 source cells, polarization started around *t* = 200 min (St. 11.5) (Fig. 2, F and G). With some delay after this polarization in cells bordering Wnt11 source cells, the intensity of GFP-Pk3 in surrounding cells became more apparent. Its orientation became aligned, and its fluctuations decreased (Fig. 2, H and J). Thus, once polarity was established in cells bordering the Wnt11 source, the polarity in surrounding cells became more apparent and their polarity direction became stabilized. This observation can be explained by a combination of the two functions of Wnt11. The bias-free function generates fluctuating polarity, and the directional function at the interface with the Wnt11 source gradually fixes the polarity of the fluctuating cell population.

In addition to these processes, we also observed another aspect of GFP-Pk3 polarization. After the direction of PCP was once stabilized, polarization of GFP-Pk3 was propagated distally without undergoing Wnt bias–free polarization in areas distal to the cells shown above (Fig. 2I). This suggests that PCP can propagate distally once stable PCP is established (Fig. 2K).

### Polarity is generated via reciprocal interaction between Wnt11 and core PCP components

**Fig. 3.**
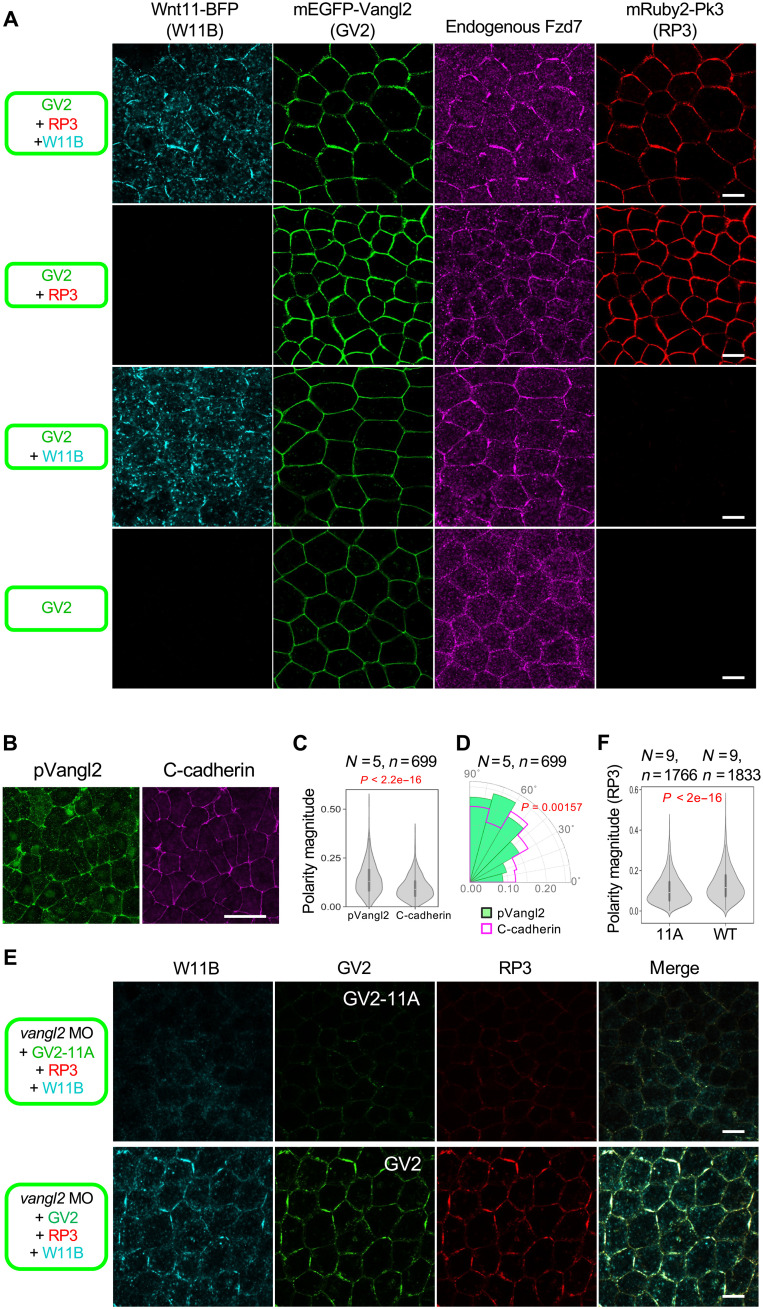
Localization of core PCP components in bias-free polarization by ubiquitous Wnt11. (**A**) Localization of core PCP components in the absence or presence of Wnt11. Components indicated at the left sides were expressed in *Xenopus* embryos by injecting mRNAs into ventral blastomeres at the four-cell stage. Coexpression of Wnt11-BFP (W11B) polarized membrane localization of GV2, RP3, and endogenous Fzd7 (first low). In addition, W11B colocalizes with these core PCP components. In the absence of W11B, membrane localization of GV2 was increased by coexpressed RP3, but its membrane localization was uniform. (**B** to **D**) Phosphorylated Vangl2 (pVangl2) is polarized in the neural plate. (B) pVangl2 staining at St. 14. C-cadherin was used as a membrane marker without polarization. (C) Polarity magnitude of pVangl2 is significantly higher than that of C-cadherin. (D) The polarity angle of pVangl2 and C-cadherin. [(C) and (D)] Statistical analyses were performed with the Kuiper two-sample test for polarity angle (D). Polarity magnitude and polarity angle were quantified with the principal components analysis (PCA) method using QuantifyPolarity2.0 (*49*). (**E** and **F**) Phosphorylation of Vangl2 is required for polarized localization of core PCP components and Wnt11. (E) Components indicated at the left sides were expressed as shown in (A). Substitution of GV2 with a phospho-deficient form (GV2-11A) leads to reduction of all observed components. (F) Quantification of PCA magnitude indicates a significant loss of polarization with GV2-11A, compared with GV2. Both GV2 and GV2-11A mRNAs are resistant to the MO. mRNAs and *vangl2* MO were injected into the animal pole region of a ventral blastomere at the four-cell stage, as indicated. Amounts of mRNAs/MOs (in nanograms per embryo): *mEGFP-vangl2* (WT and 11A), 0.10; *mRuby2-pk3*, 0.20; *wnt11-BFP*, 1.0; and *vangl2* MO, 21. Scale bars, 20 μm [(A) and (E)] and 50 μm (B). Numbers of analyzed embryos (*N*) and cells (*n*) were as indicated. Representative data from two independent experiments are presented.

In Wnt bias–free polarization, one of the main questions is how the polarization of molecules is generated without directional cues. To address this question, we examined spatial patterns of Wnt11 and core PCP components under conditions in which Wnt11 is not provided by distant source cells, but is expressed only in reporter cells. To visualize Wnt11, Vangl2, and Pk3, we used W11B, monomeric enhanced GFP (EGFP)–tagged Vangl2 (GV2), and mRuby2-tagged Pk3 (RP3), respectively. We also examined the spatial pattern of endogenous Fzd7 by immunostaining. Because stability of endogenous Fzd7 was increased simply by overexpressing Vangl2 in the same cells (figs. S3, D and E, and S7, A and B), we expressed GV2 in all experiments. First, we confirmed that addition of these tags did not perturb cell polarization because coexpression of W11B, GV2, and RP3 can polarize all these components, as well as endogenous Fzd7 (Fig. 3A, first row). As predicted, in the absence of W11B, the polarity of GV2, Pk3, and Fzd7 disappeared, showing that their polarity is dependent on Wnt11 (Fig. 3A, second row). On the other hand, Wnt11 reduced mean Pk3 levels in a dose-dependent manner, but at the same time, an intermediate dose of Wnt11 accumulated locally and polarized Pk3, indicating that appropriate levels of Wnt11 expression are important for polarization (fig. S4A). In the absence of RP3, polarity of GV2 almost disappeared, but Wnt11 and endogenous Fzd7 accumulated together with slight polarity (Fig. 3A, third and fourth rows), suggesting that Pk3 enhances Wnt11-dependent polarity. These results suggest that cell polarity can be established without directional cues, but only through reciprocal interaction between Wnt11 and core PCP components, including Vangl, Pk, and Fzd.

### Coexistent phosphorylated and nonphosphorylated states of Vangl2 are essential for polarization

To better understand the molecular mechanism of the bias-free function of Wnt11, we focused on downstream events of Wnt11 signaling. Vangl phosphorylation at the N-terminal region, which leads to Vangl2 stabilization by avoiding ubiquitination (*45*) and is essential for PCP (*27*, *46*–*48*), is induced by Wnt5a in the context of PCP formation in mammalian development (*27*, *47*). Immunostaining of phosphorylated Vangl2 (pVangl2) by anti-pVangl2 antibody (see fig. S5, A to E, for specificity) showed that pVangl2 staining preferentially accumulated at cell boundaries along the mediolateral direction of embryos (Fig. 3B), highly correlated with endogenous Wnt11 staining (fig. S5, F to H) and overexpressing W11B (fig. S5I). Furthermore, overexpression of Wnt11 increased pVangl2 staining in the animal cap region (fig. S5, A to C). These results suggest that Vangl2 phosphorylation is involved in Wnt11-induced PCP formation in *Xenopus* embryos.

To examine whether Vangl2 phosphorylation is required for the bias-free function of Wnt11, we replaced Vangl2 with mEGFP-tagged phospho-deficient Vangl2 (GV2-11A) in *Xenopus* embryos, under conditions in which endogenous Vangl2 was depleted by *vangl2*-specific MO. GV2-11A impaired the polarization of RP3 (Fig. 3, E and F), showing that phosphorylation of Vangl2 is crucial for Wnt bias–free polarization.

**Fig. 4.**
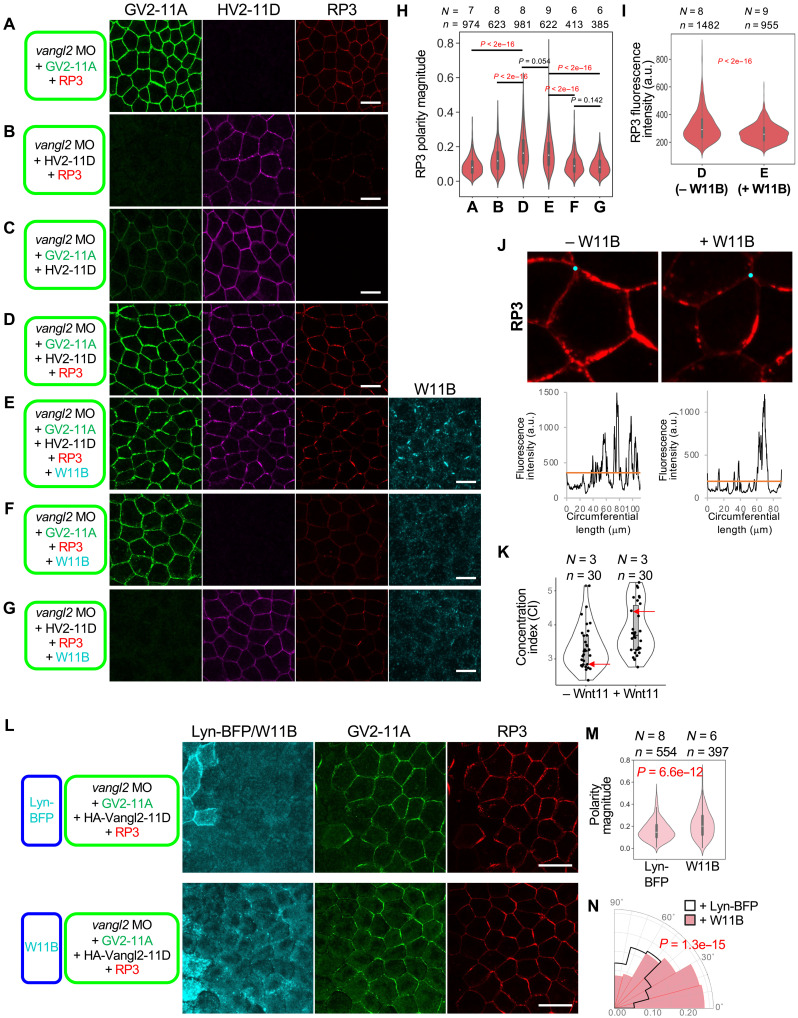
Reconstitution of cell polarity based on phosphorylated and unphosphorylated states of Vangl2. (**A** to **G**) A combination of Vangl2-11A, Vangl2-11D, and Pk3 is sufficient for self-polarization with or without Wnt11. Components indicated at the left sides were expressed as shown in Fig. 3A. Under all conditions, endogenous Vangl2 was knocked down with *vangl2* MO. mRNAs and *vangl2* MO were injected into the animal pole region of a ventral blastomere at the four-cell stage, as indicated. (**H**) Quantification of polarity magnitude of mRuby2-Pk3 shown in [(A) to (G)]. Polarity magnitude was quantified with the PCA method using QuantifyPolarity2.0 (*49*). (**I**) Quantification of RP3 on cell membranes. Conditions of –Wnt11 (D) and +Wnt (E) were compared. (**J** and **K**) Coexpression of Wnt11 concentrates Pk3 around cell circumference. (J) Representative images of RP3 in the absence (D) or presence (E) of Wnt11. Graphs show circumferential plots of RP3 (counterclockwise, starting from points indicated with cyan dots), with mean values of RP3 intensity of each cell (orange lines). (K) Concentration index (CI). CIs were calculated as follows: CI = (circumferential length of the cell)/(length with signal intensity above the mean circumferential intensity of the cell). Arrows indicate examples shown in (J). Coexpression of Wnt11 significantly increased CI. (**L** to **N**) Wnt11 overexpressed in an adjacent region can further polarize and direct Pk3. mRNAs and *vangl2* MO were injected into the animal pole region of ventral blastomeres at the four-cell stage as indicated, as in rPCP. Statistical analyses were performed with the Kuiper two-sample test for polarity angle (N). Numbers of embryos (*N*) and numbers of cells (*n*) are as indicated. Amounts of mRNAs/MOs (in nanograms per embryo): *mEGFP-vangl2-11A*, 0.050; *HA-vangl2-11D*, 0.025; *mRuby2-pk3*, 0.10; *wnt11-BFP*, 0.50; and *vangl2* MO, 21. Scale bars, 20 μm [(A) to (G)] and 50 μm (F). Representative data from two independent experiments are presented.

Next, to test the sufficiency of Vangl2 phosphorylation for Wnt11-induced polarization, we examined whether a phosphomimetic form of Vangl2, HA-Vangl2-11D (HV2-11D), can promote RP3 polarization in the absence of Wnt11 under Vangl2-depleted conditions with *vangl2* MO. As a control, GV2-11A was also used. Quantification with polarity magnitude (*49*) showed that neither HV2-11D nor GV2-11A can induce RP3 polarization (Fig. 4, A, B, and H). Notably, coexpression of GV2-11A and HV2-11D led to polarization of RP3 (Fig. 4, D and H). Under this condition, GV2-11A and HV2-11D were also polarized in a Pk3-dependent manner (Fig. 4, C and D). Thus, concurrent phosphorylated and nonphosphorylated states of Vangl2 appear important for polarization.

Unexpectedly, when W11B was coexpressed with GV2-11A, HV2-11D, and RP3 (Fig. 4E), overall, RP3 levels were reduced (Fig. 4I), but it became more concentrated on cell membranes (Fig. 4, J and K), although polarity magnitude was not significantly different (Fig. 4H). This effect is not due to phosphorylation of Vangl2, because neither GV2-11A nor GV2-11D can be further phosphorylated by W11B. Thus, in addition to effects via Vangl2 phosphorylation, Wnt11 affects localization of core PCP components independently of this phosphorylation reaction.

Last, we confirmed that coexistence of phosphorylated and nonphosphorylated Vangl2 is essential not only for Wnt bias–free polarization, but also for the establishment of Wnt bias–driven PCP, as shown in rPCP (Fig. 2) (*24*). When W11B was expressed in cells adjacent to those expressing GV2-11A, HV2-11D, and RP3 (Fig. 4L), the polarity angle of RP3 was directed toward the Wnt11 source, and the polarity magnitude of RP3 was also significantly increased (Fig. 4, M and N). Together, these results suggest that both Wnt11-induced phosphorylated and nonphosphorylated states of Vangl2 are important for the establishment PCP in *Xenopus* embryos. At the same time, these results also suggest that Wnt11 contributes to PCP formation independently of this phosphorylation reaction.

### Phosphorylated Vangl2 is polarized on the side opposite Pk3

**Fig. 5.**
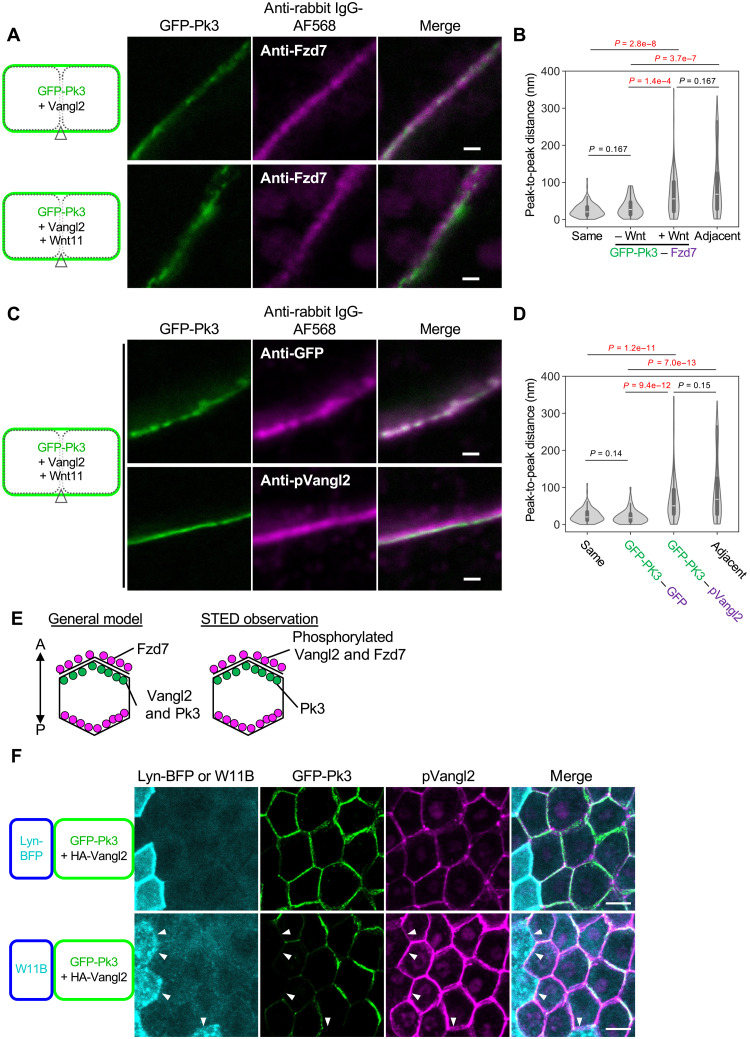
Analysis of pVangl2 in polarized cells by super-resolution microscopy (STED). (**A**) GFP-Pk3 and endogenous Fzd7 in the absence or presence of Wnt11 (not polarized or polarized, respectively) were observed by STED microscopy. (**B**) Peak-to-peak distance (PPD) between Fzd7 and GFP-Pk3 shown in (A). For comparison, PPDs between membrane traces on the same cells and on adjacent cells are shown as “same” and “adjacent,” respectively (see fig. S6C). Data from three embryos, 15 cell boundaries, 67 lines (–Wnt) and four embryos, 18 cell boundaries, 76 lines (+Wnt) are presented. (**C**) STED observations of GFP-Pk3 and phosphorylated Vangl2 in cells polarized by overexpression of Wnt11. GFP-Pk3 and immunostaining with anti-GFP or anti-pVangl2. Anti-GFP staining was used as a control that overlapped with GFP fluorescence. (**D**) PPDs between GFP-Pk3 and anti-pVangl2 staining shown in (C). Data from three embryos, 14 cell boundaries, 74 lines (anti-GFP) and three embryos, 16 cell boundaries, 90 lines (anti-pVangl2) are presented. (**E**) Localization of core PCP components based on STED microscopy. Our STED analyses suggest that phosphorylated Vangl2 localizes on the opposite side from Pk3, possibly the same side as Fzd7. Because Pk3 preferentially binds to nonphosphorylated Vangl2 (*50*), we speculate that nonphosphorylated Vangl2 is on the same side as Pk3. (**F**) pVangl2 localized to cell membranes where GFP-Pk3 was reduced by Wnt11 in rPCP. Components indicated at the left side were expressed as shown in Fig. 3A. All STED pictures in Fig. 5 and fig. S6 are shown under the same condition for brightness/contrast. mRNAs were injected into animal pole regions of ventral blastomeres at the four-cell stage, as indicated. Amounts of mRNAs (in nanograms per embryo): *GFP-pk3*, 0.20; *vangl2*, 0.10; *HA-vangl2*, 0.10; *wnt11*, 0.25; *wnt11-BFP*, 0.50; and *lyn-BFP*, 0.050. Scale bars, 500 nm [(A) and (C)] and 20 μm (F). Representative data from two independent experiments are presented.

Vangl2 phosphorylation is dependent on Fzd but is inhibited by Pk (*46*, *50*). Furthermore, this phosphorylation inhibits interaction between Vangl2 and Pk3 in *Xenopus* embryos (*50*). Although Vangl localizes on Pk-rich cell membranes in polarized cells, these findings imply that pVangl2 does not. Thus, we minutely examined localization of pVangl2 at the cell boundary using super-resolution stimulated emission depletion (STED) microscopy, which was able to resolve localization of membrane tracers expressed in adjacent cells (fig. S6, A to C), to analyze Wnt bias–free polarization (see Fig. 2D and fig. S4). STED observations showed that Wnt11 segregates Pk3 and Fzd7 staining (Fig. 5, A and B), corresponding to their localization on opposite sides at the cell boundaries of polarized cells. Notably, under this condition, pVangl2 staining was segregated from GFP-Pk3 (Fig. 5, C and D, and fig. S6D), suggesting that Vangl2 phosphorylation specifically occurs on the Fzd-side in polarized cells (Fig. 5E). Consistent with this hypothesis, at the cell membrane facing Wnt11-expressing cells, pVangl2 was clearly observed, along with disappearance of GFP-Pk3 (Fig. 5F).

### Wnt11 induces assembly of phosphorylated Vangl2 and Fzd7 on the same cell membrane

**Fig. 6.**
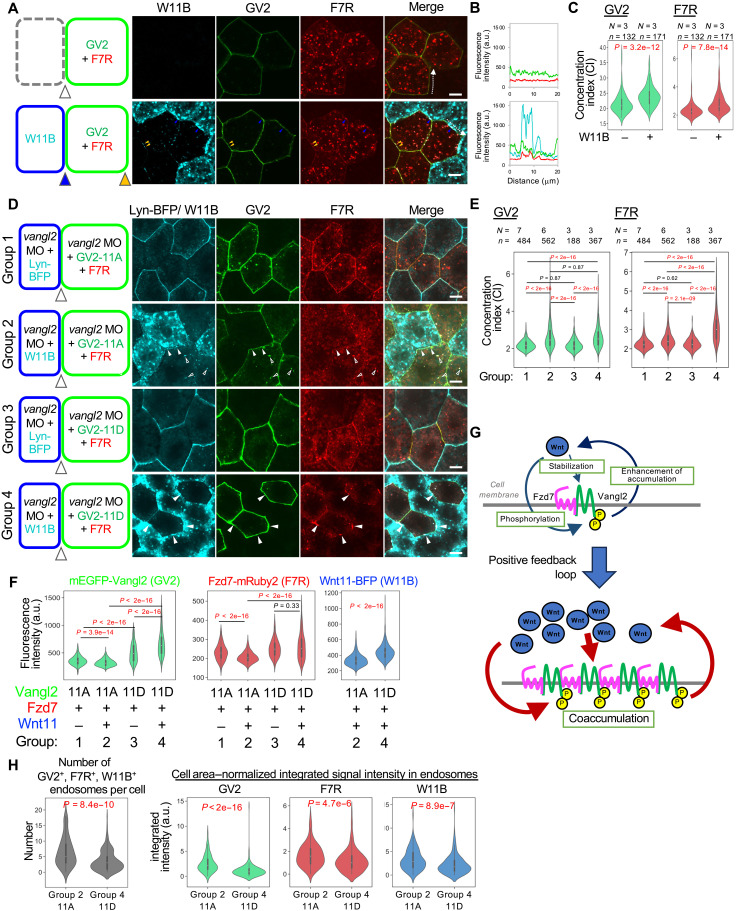
Cis-assembly of Vangl2 and Fzd7 induced by Wnt11, involving Vangl2 phosphorylation states. The design of each experiment is shown in (A) and (D). (**A**) Wnt11 induces cis-assembly of Vangl2 and Fz7. Cis-assembly is frequently observed on outer boundaries facing W11B-expressing cells (blue arrowheads), but also on other outer boundaries (orange arrowheads). (**B**) Intensity profiles of each signal along the outer cell boundaries shown by dotted arrows in (A) (cyan, W11B; green, GV2; red, F7R). (**C**) Concentration indices (CIs). (**D**) Effects of Vangl2 phosphorylation states on cis-assembly with Fzd7. Open arrowheads, vesicles containing GV2-11A, F7R, and W11B, indicating endosomal internalization. Closed arrowheads, cis-assembly of GV2-11D and F7R on cell membranes. (**E**) CIs. (**F**) Quantification of tagged proteins at outer cell boundaries of GV2- and F7R-expressing cells [arrowheads in each schematic image in (D)]. Numbers of embryos (*N*) and numbers of cell boundaries (*n*): Group 1, *N* = 7, *n* = 485; Group 2, *N* = 6, *n* = 562; Group 3, *N* = 3, *n* = 188; and Group 4, *N* = 3, *n* = 367. (**G**) Schematic representation based on (A) to (F). (**H**) Phospho-deficient Vangl2 was highly internalized compared with phosphomimetic Vangl2 in the presence of Wnt11. The same experiments as shown in (D) were performed with an increased number of embryos. Numbers of embryos (*N*) and numbers of cells (*n*): Group 2, *N* = 10, *n* = 122; Group 4, *N* = 9, *n* = 199. mRNAs and *vangl2* MO were injected into the animal pole region of ventral blastomeres at the four-cell stage. Amounts of mRNAs/MOs (in nanograms per embryo): *mEGFP-vangl2*, 0.20; *mEGFP-vangl2-11A*, 0.10; *mEGFP-vangl2-11D*, 0.10; *fzd7-mRuby2*, 0.20 [(A) to (C)], 0.050 (D), and 0.10 [(E), (F), and (H)]; *wnt11-BFP*, 1.0 [(A) to (C)] and 0.50 [(D) to (F) and (H)]; *lyn-BFP*, 0.050; and *vangl2* MO, 42 (D) and 28 [(E), (F), and (H)]. Scale bars, 10 μm. Representative data from two independent experiments are presented.

Our analysis using STED microscopy suggests that Wnt11 induces assembly of pVangl2 with Fzd7 on the same membrane. Because overexpressed Vangl2 increased Fzd7 amounts on the same membrane (figs. S7, C to F, and S8), we asked whether and how Wnt11 influences Fzd7 via phosphorylation of Vangl2 on the same membranes. For this purpose, mRuby2-tagged Fzd7 [Fzd7-mRuby2 (F7R)] was expressed with GV2, GV2-11A, or a phosphomimetic form, GV2-11D, in a group of cells. Their outer boundaries facing neighboring cells that did not express GV2 or F7R were analyzed under conditions with or without W11B. With GV2-11A and GV2-11D, the effect of endogenous Vangl2 was depleted with a *vangl2*-specific MO (Fig. 6D). While GV2 and F7R exhibit uniform localization along the outer boundaries (Fig. 6A and fig. S9A), they assembled with W11B when W11B was expressed in adjacent cells (Fig. 6, A and B, and fig. S9A). Thus, Vangl2 and Fzd7 can coaccumulate on the same membrane while interacting with Wnt11 [Fig. 6C; the degree of assembly of Vangl2, Fzd7, and W11B is evaluated with the concentration index (CI)]. To distinguish these assemblies on the same membranes from those bridging opposing membranes, the former are referred to hereafter as cis-assemblies and the latter as trans-assemblies.

Assembly of Vangl2 and Fzd7 with Wnt11 was also observed even with GV2-11A (Fig. 6, D and E, and fig. S9B; Group 1 versus 2), suggesting that Wnt11 can recruit Vangl2 and Fzd7 even when Vangl2 is not phosphorylated. On the other hand, this assembly was more continuous and intense with GV2-11D (fig. S10, B and D), and GV2-11D concentrated F7R more than GV2-11A did (Fig. 6, D and E; Group 2 versus 4). Thus, Vangl2 phosphorylation further promotes Wnt11-dependent cis-assemblies. Because this phosphorylation is dependent on Wnt/Fzd signaling (*27*, *50*), our results suggest that Vangl2 phosphorylation and Wnt11/Fzd7 signaling promote one another mutually, forming a positive feedback loop that enhances assembly of Vangl2, Fzd7, and Wnt11 (Fig. 6G). In addition, the abundance of GV2-11D on outer boundaries was higher than that of GV2-11A, probably due to differences of transport efficiency from the endoplasmic reticulum (ER) to the cell membrane, as previously reported (*45*), and those of F7R and W11B were also higher with Vangl2 phosphorylation (Fig. 6F and figs. S7, C to F, and S8; Group 1 versus 3 and Group 2 versus 4). Thus, Vangl2 phosphorylation may increase the abundance of Vangl2 itself, as well as of Fzd7 and Wnt11 at the outer border (Fig. 6G).

Abundance of Vangl2, Fzd7, and W11B on outer boundaries appears to show additional functions of Wnt11 in cis-assembly. GV2-11A was decreased by W11B (Fig. 6F; Group 1 versus 2), whereas GV2-11D was increased (Fig. 6F; Group 3 versus 4). Wnt11 also reduced the amount of F7R on the same membranes as GV2-11A (Fig. 6F; Group 1 versus 2). Given that GV2-11A and GV2-11D may not be further phosphorylated, this result suggests that some Wnt11 function other than inducing Vangl2 phosphorylation regulates the stability of Vangl2 and Fzd7, depending on the phosphorylation states of Vangl2. Thus, Wnt11 not only increases phosphorylation of Vangl2, but also further stabilizes phosphorylated Vangl2, thereby promoting assembly of Wnt11, Fzd7, and Vangl2.

Intracellular puncta positive for GV2, F7R, and Wnt11 were significantly more abundant with GV2-11A than with GV2-11D (Fig. 6H and fig. S10E). Considering that intracellular Wnt11 was only taken up from outside the cell, these results suggest that nonphosphorylated Vangl2 tends to be removed with Fzd7 and Wnt11 from the cell membrane via endocytosis (fig. S10I). Furthermore, when RP3 was expressed with W11B and GV2-11A, RP3 on cell membranes was reduced with GV2-11A by W11B, and their endosome-like puncta were increased by W11B (fig. S10F, same condition as Fig. 4, A and F, and fig. S10H). In addition, in embryos in which Wnt bias–free polarization was observed, GFP-Pk3 was often found in endosomes that were negative for pVangl2 staining (fig. S10G). Thus, it is plausible that Pk3, nonphosphorylated Vangl2, and Fzd7 are coupled so as to be internalized into cells upon Wnt11 stimulation (fig. S10I). This Wnt11/Fzd7-mediated internalization machinery may result in exclusion of Pk3 and nonphosphorylated Vangl2 from membrane regions enriched in Fzd7 and pVangl2.

### A combination of regulatory loops leads to asymmetric arrangement of core PCP components

**Fig. 7.**
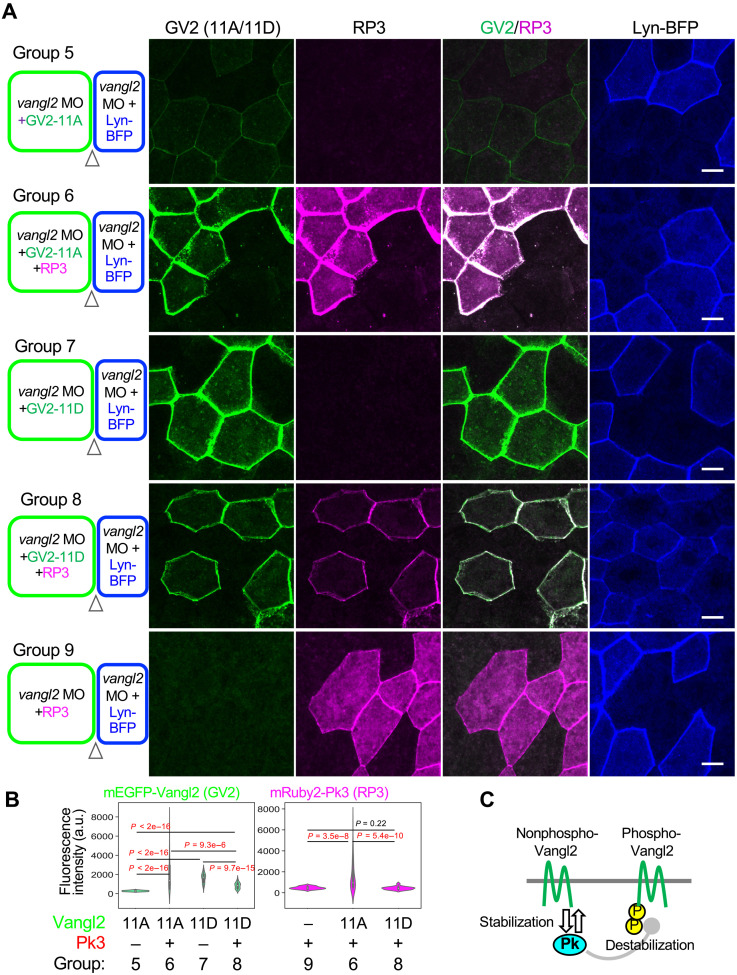
Reciprocal stabilization of Pk3 and nonphosphorylated states of Vangl2. (**A** and **B**) Effects of Vangl2 phosphorylation states with Pk3 on the same membrane. (A) The design of each experiment is shown at the right side. Five experimental groups (Group 5 to 9) were examined as indicated. (B) Outer cell boundaries of GV2 and F7R expressing cells, where both are on the same membrane, are quantified (arrowheads in schematic illustrations) for amounts of tagged proteins. Numbers of embryos (*N*) and numbers of cell boundaries (*n*): Group 5, *N* = 7, *n* = 112; Group 6, *N* = 4, *n* = 55; Group 7, *N* = 5, *n* = 94; Group 8, *N* = 7, *n* = 55; and Group 9, *N* = 5, *n* = 46. (**C**) Schematic illustration of stabilization/destabilization of Vangl2-Pk3 cis-complexes switched with phosphorylation states of Vangl2. mRNAs and *vangl2* MO were injected into animal pole regions of ventral blastomeres at the four-cell stage, as indicated. Amounts of mRNAs/MOs (in nanograms per embryo): *mEGFP-vangl2-11A*, 0.10; *mEGFP-vangl2-11D*, 0.10; *mRuby2-pk3*, 0.20; *lyn-BFP*, 0.050; and *vangl2* MO, 42. Scale bars, 10 μm. Representative data from two independent experiments are presented.

Because our STED analysis suggests that Pk3 is mostly localized on the opposite side of pVangl2, we examined whether phosphorylation of Vangl2 impairs the stability of Pk3 on the same membrane. As predicted, GV2-11A significantly increased mRuby2-Pk3 (RP3), whereas GV2-11D did not (Fig. 7, A and B, right). On the other hand, RP3 increased GV2-11A but decreased GV2-11D (Fig. 7, A and B, left). Thus, Pk3 and nonphosphorylated Vangl2 appear to stabilize each other on the opposite side from pVangl2 and Fzd7 (Fig. 7C).

**Fig. 8.**
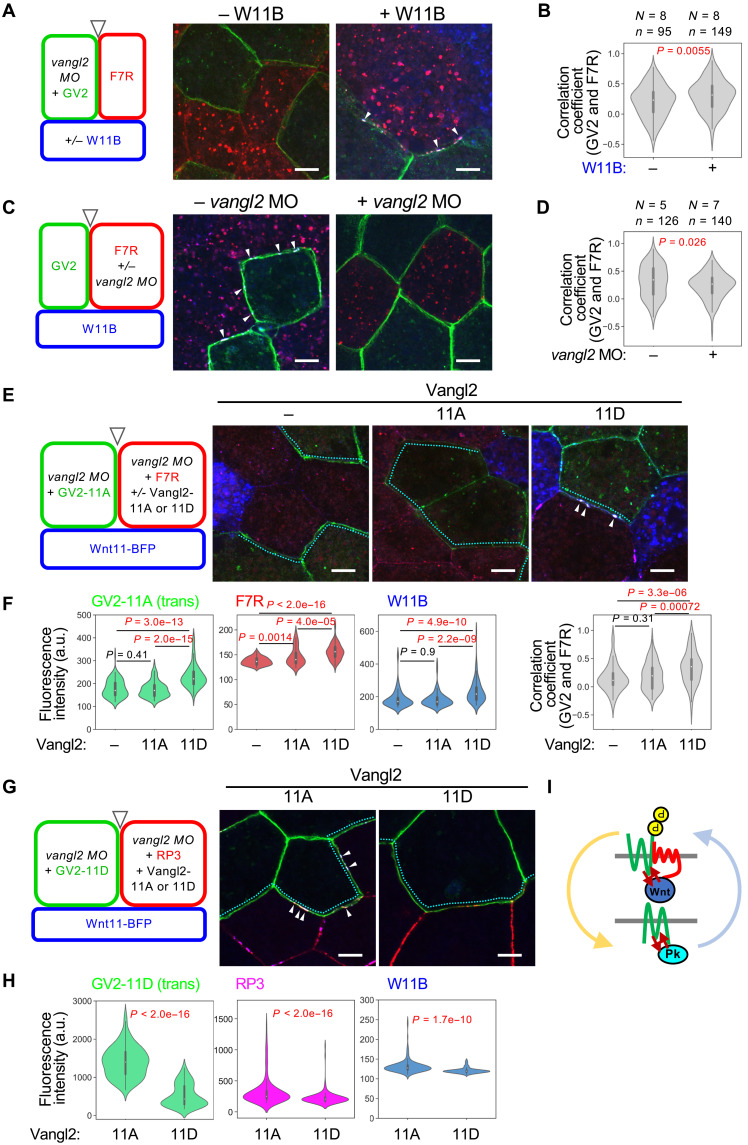
Trans-assembly of core PCP components by Wnt11. The design of each experiment is shown in (A), (C), (E), and (G). (**A**) Wnt11 induces trans-assembly of Vangl2 and Fzd7. Arrowheads, trans-assemblies of GV2 and F7R with W11B. (**B**) Correlation coefficients between GV2 and F7R in (A). (**C**) Trans-assemblies of GV2 and F7R (arrowheads) were inhibited by *vangl2* MO into the F7R side. (**D**) Correlation coefficients of GV2 and F7R on opposing membranes in (C). (**E**) Vangl2-11D on the same membrane as Fzd7 can specifically increase Vangl2-11A on the opposite side. (**F**) Boundaries between GV2-expressing and F7R-expressing cells in (E) were quantified (cyan dotted lines). *Cis*-Vangl2 (−), *N* = 4, *n* = 97; 11A, *N* = 4, *n* = 81; and 11D, *N* = 6, *n* = 94. (**G**) Vangl2-11A on the same membrane as Pk3 can specifically increase Vangl2-11D on the opposite side. (**H**) Boundaries between GV2-expressing and RP3-expressing cells in (G) were quantified (cyan dotted lines). 11A, *N* = 8, *n* = 177; 11D, *N* = 4, *n* = 110. (**I**) Schematic illustration of reciprocal stabilization of opposing phosphorylated and nonphosphorylated Vangl2, corresponding to Fzd7 and Pk3 sides, respectively. mRNAs for GV2, GV2-11A/D, F7R, Vangl2-11A/D, and *vangl2* MO were injected into ventral blastomeres, and mRNA for W11B was injected into dorsal blastomeres at the four-cell stage, as indicated. Amounts of mRNAs/MOs (in nanograms per embryo): *mEGFP-vangl2*, 0.10 [(A) and (B)] and 0.20 [(C) and (D)]; *mEGFP-vangl2-11A*, 0.10; *mEGFP-vangl2-11D*, 0.20; *fzd7-mRuby2*, 0.10 [(A) and (B)], 0.20 [(C) and (D)], and 0.050 (E); *mRuby2-pk3*, 0.20; *wnt11-BFP*, 1.0 [(A), (B), (E), and (F)] and 2.0 [(C) and (D)]; *vangl2-11A/D*, 0.10 (E) and 0.050 (F); and *vangl2* MO, 11 [(A) and (B)], 21 [(C) and (D)], 57 (E), and 42 (F). Scale bars, 10 μm. Representative data from two independent experiments are presented.

In PCP, core PCP components are asymmetrically localized across cell boundaries, i.e., asymmetry with Pk and Fzd (see Fig. 5E), and are likely to accumulate in a trans-configuration, bridging adjacent cells (*33*–*35*, *51*). We found that overexpressed W11B promoted trans-assembly of GV2, F7R, and W11B (Fig. 8, A and B, and fig. S11A). When endogenous Vangl2 was knocked down on the side of F7R, GV2-F7R trans-assembly was reduced (Fig. 8, C and D, and fig. S11B), indicating that Vangl2 on the Fzd side is prerequisite for Vangl2-Fzd7 trans-assembly. To examine whether phosphorylation of Vangl2, which is crucial for cis-assembly with Fzd7, is also important for trans-assembly, nontagged Vangl2-11A or Vangl2-11D was expressed on the same side as F7R under conditions in which GV2-11A was expressed on the opposite side. *Cis*-Vangl2-11D significantly increased the level of GV2-11A and F7R, compared to controls without exogenous Vangl2 and Vangl2-11A, suggesting that cis-assembly of phosphorylated Vangl2 and Fzd7 increases the level of nonphosphorylated Vangl2 on the opposite side (Fig. 8, E and F, and fig. S11C).

In turn, we examined whether Vangl2-Pk3 cis-interaction affects the level of phosphorylated Vangl2 on the opposite side. In this case, nontagged Vangl2-11A or Vangl2-11D was expressed on the same side as RP3 under conditions in which GV2-11D was expressed on the opposite side. We found that Vangl2-11A increased the GV2-11D level more than Vangl2-11D did (Fig. 8, G and H, and fig. S11D). These results indicate that the two types of cis-interactions either involving Fzd7 or Pk3 form on different sides of the cell boundary and stabilize formation of one another through trans-interaction. Given that Wnt11/Fzd7/pVangl2-mediated cis-assembly is amplified by a positive feedback loop, this amplification may spill over into the trans-interaction and even to Vangl2-Pk3 cis-interaction, leading to coordinated amplification of symmetry breaking across the cell boundary (Fig. 8I).

## DISCUSSION

It is still debatable whether the concentration gradient of Wnt ligands serves as a directional cue for PCP. In vertebrates, some studies support the idea that Wnt functions as a signaling molecule that provides directional cues (*27*, *52*). For instance, Vangl2 phosphorylation, which is induced by Wnt5a through Ror2, exhibits a gradient along the proximo-distal axis in the limb bud (*27*). Although this suggests that a Wnt5a signaling gradient is generated along the proximo-distal axis of the limb bud, it remains unclear whether Wnt ligands themselves simply form concentration gradients during PCP formation. Furthermore, it is also puzzling how each cell in a gradient senses the direction of the surrounding Wnt gradient and establishes its own polarity.

In this study, we found that Wnt11 does not form simple gradients as previously predicted (Fig. 1, A to C, and fig. S1, D and E), but accumulates along the polarity of PCP in the *Xenopus* neural plate. Accumulated Wnt11 is colocalized with core PCP components, suggesting local, reciprocal interaction. In the presence of core PCP components, Wnt11 can induce its own polarization and that of core PCP components, even if Wnt11 is not provided as a gradient (Figs. 3A and 8I). Thus, bias-free Wnt11 and core PCP components can autonomously generate cell polarity without any directional information and Wnt11 can be recognized as a soluble component that interacts tightly with a core PCP component, i.e., a “soluble” core PCP component. Our observation of Wnt bias–free polarization is based on gain-of-function experiments, but a previous study showing that uniform expression with mRNA injection can partially rescue zebrafish *wnt11* mutants suggests that Wnt11 acts in a similar manner under physiological condition (*25*). Another study also suggests a permissive function of *wnt11* in zebrafish mechanosensory hair cell orientation (*53*). On the basis of our results, we propose a model in which PCP is self-organized in a bottom-up manner through local and reciprocal interaction between Wnt11 and core PCP components, by forming asymmetric complexes (Fig. 8I).

**Fig. 9.**
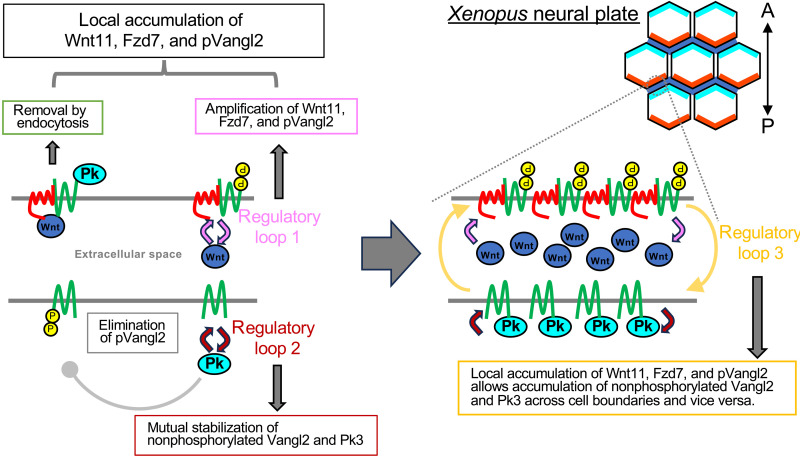
Model of asymmetric complex formation by Wnt11. Wnt11 promotes phosphorylation of Vangl2 (fig. S5, A to C), likely via Fzd7, and further stabilizes cis-complexes of pVangl2 and Fzd7 (Fig. 6, D to F). In turn, these cis-complexes accumulate more Wnt11 (Fig. 6, D and F) to form a positive regulatory loop (1) (Fig. 9). On the other hand, phosphorylation of Vangl2 may be inhibited in the presence of Pk3 (fig. S5, A to C). Phospho-deficient Vangl2 or Vangl2 with Pk3 tend to be removed with Fzd7 and Wnt11 by endocytosis (Fig. 6, D to H, and fig. S10, E to I), consistent with dose-dependent reduction of GFP-Pk3 by Wnt11 (fig. S4). This mechanism could lead to a coupled removal of Fzd7 and Pk3 on the same membrane. When abundance of Pk3 exceeds that of Fzd7, Pk3 and nonphosphorylated Vangl2 mutually stabilize each other, and Pk3 and pVangl2 are mutually inhibitory (Fig. 7), forming another regulatory loop (2). Superficially, this loop does not involve Wnt11, consistent with the absence of Fzd7 on the membrane as a result of the coupled removal of Fzd7 and Pk3. In addition to these regulatory loops involving cis-complexes, the pVangl2-Fzd7 side and the non-pVangl2-Pk3 side preferentially face each other (regulatory loop 3), likely through trans-complex formation bridging adjacent cells (Fig. 8).

As part of the mechanisms involved in this self-organizing PCP formation, we revealed local and reciprocal interactions between Wnt11 and core PCP components. We showed that Wnt11, which can induce Vangl2 phosphorylation, accumulates pVangl2 on the same cell membranes as Fzd7, and, conversely, Vangl2 phosphorylation promotes coaccumulation of pVangl2 and Fzd7 with Wnt11 (Fig. 6, D to G). Thus, phosphorylated Vangl2 and Wnt11 accumulate one another in a mutual regulatory loop, accounting for the polarized localization of Wnt11, Fzd7, and pVangl2 (Fig. 6, A to D). We propose that continuation of this regulatory loop amplifies local concentrations of Wnt11, Fzd7, and pVangl2 on the cell membrane. In addition to this loop, we showed that two other mutual regulatory interactions, in which each component reciprocally activates the other, are involved. One is a cis-interaction between nonphosphorylated Vangl2 and Pk3, which does not necessarily involve Wnt11 (Fig. 7). The other is a trans-interaction between phosphorylated and nonphosphorylated Vangl2, located on opposing cell membranes (Fig. 8). Through the trans-interaction, the local accumulation of Wnt11, Fzd7, and pVangl2 preferentially coordinates nonphosphorylated Vangl2 at the opposing plasma membrane site, and the interaction between nonphosphorylated Vangl2 and Pk3 also results in the coordination of Pk3 with nonphosphorylated Vangl2 (Fig. 9). Thus, once Wnt11, Fzd7, and pVangl2 stochastically accumulate on the plasma membrane, nonphosphorylated Vangl2 and Pk3 preferentially align on the opposite side, resulting in asymmetry across the plasma membrane. In addition to the combinatory mechanism of the three regulatory loops, the inhibitory mechanism of Pk3 against pVangl2 (fig. S5, A to C, and Fig. 7C) appears to eliminate pVangl2 from the Pk3 side. Because coincorporation of Wnt11 with Fzd7 and nonphosphorylated Vangl2 or Pk3 was frequently observed (Fig. 6, D to H, and fig. S10, E to I), Wnt11-mediated endocytosis seems to be involved in the elimination of Pk3 from the Fzd7 side and vice versa. On the other hand, we acknowledge a limitation of this study in that our model is primarily based on quantitative analyses of fluorescence imaging. To further examine this model, future studies will be required to identify, through biochemical approaches, the core PCP protein complexes formed on each side of the cell membrane and to quantitatively assess the stability of each protein at the plasma membrane.

The importance of cell-bridging interactions of core PCP components, including Vangl/Stbm, Fzd, and Celsr/Fmi, for PCP has also been reported in both mice and *Drosophila* (*33*–*35*, *51*). Because phosphorylation of Vangl in vertebrates (and Vang/Stbm in *Drosophila*) has been described as an important event in PCP (*27*, *46*–*48*), it is an interesting question whether the intercellular interaction mediated by phosphorylated and nonphosphorylated Vangl2 is also involved in many aspects of PCP formation. Furthermore, it will be important in future studies to determine whether the model demonstrated in this study, including the proposed intercellular interactions, is also applicable to other contexts of PCP formation.

We previously showed that Wnt8 proteins coassemble on heparan sulfate proteoglycan (HSPG) clusters on the cell membrane (*41*), probably contributing to the assembly of signalosome components, including Fzd, on the cell membrane. Thus, interactions between Wnt and HSPGs may influence accumulation of core PCP components and Wnt11 observed in this study. Coaccumulations of Wnt ligands and Fzd are observed in Wnt-dependent cell polarization of *Caenorhabditis elegans* (*54*). In addition, Dvl is also accumulated by Wnt in the context of PCP (*55*, *56*). In *Drosophila*, signalosome-like assemblies of PCP components have also been reported (*34*, *48*, *57*); thus, it is interesting whether the assemblies actually require Wnt ligands (*23*, *29*, *30*). Thus, we speculate that some machinery involved in signalosome formation may be used in Wnt-dependent assemblies in PCP formation.

## MATERIALS AND METHODS

### *Xenopus* embryo manipulation and microinjection

All experiments using *Xenopus laevis* were approved by the Institutional Animal Care and Use Committee, National Institutes of Natural Sciences (license numbers: 12E076, 13A187, 14A066, 15A101, 16A126, 17A063, 18A038, 19A062, 20A053, 21A078, 22A023, 23A099, and 24A073) or the Animal Experimentation Committee, Kyoto University (license numbers: M-24-3-2 and M-24-3-3). Handling of *X. laevis* and microinjections were performed using standard methods (*58*). In brief, female frogs were injected with gonadotropin (ASKA Pharmaceutical) to obtain unfertilized eggs, which were then fertilized with testis homogenates. Fertilized eggs were de-jellied in 4% cysteine (pH 7.8) and incubated in 0.1× Steinberg’s solution at 14° to 20°C. Embryos were staged according to Nieuwkoop and Faber (*59*) and Zahn *et al.* (*60*). *Xenopus* illustrations © Natalya Zahn (2022) (CC BY-NC, https://creativecommons.org/licenses/by-nc/4.0/deed.en) were obtained from Xenbase (www.xenbase.org; RRID:SCR_003280). Microinjections were performed on early-stage embryos (4- to 32-cell stages) in 3% Ficoll (Sigma-Aldrich)/modified Birth’s solution. mRNAs used in microinjections were synthesized from plasmid DNAs using an mMESSAGE mMACHINE SP6 or T7 Kit (Invitrogen) and purified with an RNeasy Micro Kit (QIAGEN). MOs were used in knockdown experiments. Unless specified in the figure legends, embryos were observed at St. 14. Amounts of injected mRNAs/MOs are described in the figure legends.

### Plasmid constructs

The following plasmids were used as templates for mRNA synthesis: pXT7moo2-GFP-Xpk3 (linearized with Xba I) (*24*); pCS2+HA-vangl2 (*24*); pCS2+wnt11-6Myc (*61*); and pCS2+mRFP1 (*62*). To generate pCS2+vangl2, the Bam HI/Xba I fragment of pCS2+HA-vangl2 was replaced with a polymerase chain reaction–amplified fragment containing the vangl2 coding sequence (CDS) to remove the HA tag. To generate pCS2+HA-vangl2-11D, the Eco RI/Eco RV fragment of pCS2+HA-vangl2 was replaced with a synthesized fragment encoding vangl2-11D (IDT). All pCS2+ constructs were linearized with Not I prior to mRNA synthesis. The following genes/CDSs were subcloned into pCSf107mT (*63*), which does not require linearization for mRNA synthesis: mEGFP-krasCT and mRuby2-krasCT (*64*); lyn-mEGFP and lyn-BFP (lyn-mTagBFP2-3HA) (*65*); wnt11 and wnt11-BFP (wnt11-GS linker-mTagBFP2) (*61*, *66*); tethered-wnt11 (wnt11-CD8TM-mCherry, in which the CDS for CD8TM-mCherry was derived from the morphotrap construct (*67*); mEGFP-vangl2 [wild type (WT), 11A, and 11D], for which the CDSs of vangl2-11A and vangl2-11D were synthesized by IDT; and fzd7 and fzd7-mRuby2 (*61*, *68*). pCSf107-mRuby2-pk3 was also used (*44*).

### Morpholino oligos

The following MOs were used in this study: *wnt11b*, CCAGTGACGGGTCGGAGCCATTGGT (*69*); *vangl2*, GAGTACCGGCTTTTGTGGCGATCCA (*70*) and CGTTGGCGGATTTGGGTCCCCCCGA (*71*); and *fzd7*, CCAACAAGTGATCTCTGGACAGCAG (*72*) and GCGGAGTGAGCAGAAATCGGCTGAT (*73*). Standard (std) MO (CCTCTTACCTCAGTTACAATTTATA) was used as a negative control.

### Fluorescence image acquisition

Fluorescence images were acquired with a spinning disk confocal microscope (Andor Dragonfly 200 combined with an Olympus IX83, objective: UPLXAPO 20×/NA 0.8) or laser-scanning confocal microscope (Leica TSC SP8 system, objective: HC PL APO 40×/NA 1.10 W CORR water immersion).

### Super-resolution microscopy

STED microscopy was carried out with a Leica TCS SP8 STED 3X FALCON system (objective: HC PL APO 100×/1.40 OIL CS2), as previously described (*41*). Because WT embryos were destroyed by STED observation due to heat generated in pigment granules, albino embryos were used for analysis. These were fixed at St. 14 and stained with Alexa Fluor 488– and/or Alexa Fluor 568–labeled secondary antibody. For STED, a 592-nm laser was used for GFP and Alexa Fluor 488, and a 775-nm laser was used for Alexa Fluor 568. STED images were processed with the “tau STED” function (LAS-X software, Leica) using fluorescence lifetime analysis to enhance separation and localization of fluorescent peaks (*74*). Peak-to-peak distance (PPD) was semiautomatically quantified using Fiji/ImageJ with the“FWHM line”plugin (Lim Soon Yew John, in the ImageJ Mailing List).

### Production of an anti-Wnt11 monoclonal antibody

We produced a mouse monoclonal antibody against *Xenopus* Wnt11 (clone no. 56-1), as described previously (*75*). Briefly, recombinant full-length *Xenopus* Wnt11 was prepared as previously described (*41*, *76*), and the antigen emulsion was injected into BALB/c mice. Treated mice were euthanized 21 days after injection, and lymphocytes were fused with SP2/0-Ag14 myeloma cells. After the cell fusion, culture supernatants were screened to confirm positive clones with a solid-phase enzyme-linked immunosorbent assay (ELISA). ELISA-positive clones were further screened by immunostaining of Wnt11-6Myc overexpressing embryos.

### Immunostaining

*Xenopus* embryos were fixed with MEMFA (0.1 M MOPS, pH 7.4, 2 mM EGTA, 1 mM MgSO_4_, and 3.7% formaldehyde). For endogenous Wnt11 staining, fixed embryos were dehydrated with EtOH and rehydrated with TBT (1× tris-buffered saline, 0.2% bovine serum albumin, and 0.1% Triton X-100). After replacement with TBT, fixed embryos were blocked with TBTS [TBT with 10% heat-immobilized fetal bovine serum (70°C, 40 min)] for 1 hour. For staining total Vangl2, antigen retrieval was performed at 95°C for 20 min in citrate-based Antigen Unmasking Solution (H-3300-250, Vector Labs) before blocking. Primary antibodies and their dilutions were as follows: anti-Wnt11 [mouse monoclonal immunoglobulin G2b (IgG2b), in-house preparation, 1/10], anti-Vangl2 (HPA027043, Sigma-Aldrich, rabbit polyclonal IgG, 1/200), anti-Fzd7 (ab64636, Abcam, rabbit polyclonal IgG, 1/200), anti-phosphorylated Vangl2-T78/S79/S82 (AP1206, ABclonal, rabbit polyclonal IgG, 1/1000), anti-C-cadherin (6B6, Developmental Studies Hybridoma Bank, mouse monoclonal IgG1, 1/50), and anti-β-catenin (33-9100, Invitrogen, ZO1-1A12, mouse monoclonal IgG1, 1/2000). Primary antibodies were diluted with Can Get Signal immunostain Immunoreaction Enhancer Solution A (NKB-501, TOYOBO) or B (NKB-601, TOYOBO) or TBTS. Secondary antibodies and their dilutions were as follows: goat polyclonal anti-mouse IgG Alexa Fluor 488 (A11001, Molecular Probes, 1/500), goat polyclonal anti-mouse IgG Alexa Fluor 647 (A21235, Molecular Probes, 1/500), goat polyclonal anti-rabbit IgG Alexa Fluor 488 (A11008, Molecular Probes, 1/500), goat polyclonal anti-rabbit IgG Alexa Fluor 555 (A21429, Molecular Probes, 1/500), and goat polyclonal anti-rat IgG Alexa Fluor 555 (A21434, Molecular Probes, 1/500). Secondary antibodies were diluted with TBTS. Embryos were incubated in antibody solution overnight at 4°C and washed with TBT 6 × 30 min after incubation with primary or secondary antibodies. Wheat germ agglutinin–conjugated with Alexa Fluor 647 (W32466, Molecular Probes) was mixed in secondary antibody solution at a dilution of 1/2000.

### Whole-mount in situ hybridization

For probe preparation, pCSf107-Xwnt11 was linearized with Bam HI for antisense probe synthesis or with Xba I for sense probe synthesis. pCSf107-Xfzd7 was linearized with Bam HI for antisense probe synthesis. Using these template DNAs, digoxigenin (DIG)–labeled RNA probes were synthesized with DIG RNA Labeling Mix (Roche, 11277073910) and T7 (for antisense probes) or SP6 (for sense probes) RNA polymerase (Roche, RPOLSP6-RO and RPOLT7-RO). Whole-mount in situ hybridization was performed manually according to the method of Harland (*77*), as previously described (*61*). For figs. S1A and S3A, staining was performed using anti-DIG-AP (Roche, 11093274910) and BM Purple (Roche, 11442074001). For fluorescence in situ hybridization (fig. S3, F and G), staining was performed using anti-DIG-POD (Roche, 11207733910) and the TSA Plus Cyanine3/Fluorescein System (Perkin Elmer, 753001KT).

### Image analysis

All measurements of signal intensity were performed with Fiji/ImageJ (v1.54f). To obtain spatial intensity profiles of endogenous Wnt11 in the neural plate (Fig. 1C and fig. S1F), intensity profiles were obtained from posterior to anterior along a 100-pixel-width (=151.67 μm) “straight line.” To evaluate the effects of knockdown experiments on endogenous factors in the neural plate (Fig. 1, F and G, and fig. S5, D and E) and because basal signal intensity varied among embryos, normalized intensities of regions of interest (ROIs) were calculated by dividing intensities of ROIs by the averaged intensity of the uninjected side for each embryo. For fig. S1B, signal intensity was normalized to the averaged Wnt11 intensity in std MO-injected embryos. Signal intensities of cell membranes were obtained by generating an intersection image of a cell segmentation mask (5- to 10-pixel width) and an image to be quantified using “Subtract” operation of the “Calculator Plus” plugin in Fiji/ImageJ. Segmentation masks were initially generated with “Cellpose” (using the model of “Cyto2” or “Cyto3”) (*78*) based on membrane marker images and subsequently corrected by “TissueAnalyzer” (*79*) in the Fiji/ImageJ plugin. For the measurement of signal intensities and correlation of factors in Figs. 4 to 8 and figs. S4, S5, S7, S8, and S10, ROIs of each cell boundary were obtained by manually tracing cell boundaries with lines 5 or 10 pixels using an iPad (Apple). Cell boundaries of interest were randomly selected as previously described (*41*). Intensity profiles were collected with an in-house Fiji/ImageJ macro (auto-line-plot.ijm), and averaged intensities and correlation coefficients among factors were calculated with an in-house Python code (4-channel-line plot.py).

For quantification of polarity angles and polarity magnitude in Figs. 1 to 4 and fig. S3, cell segmentation images were generated as described above. Polarity angles and magnitudes were quantified by “QuantifyPolarity2.0” with the principal component analysis (PCA) method (*49*). For polarity angles of GFP-Pk3 (Fig. 2E) and mRuby2-Pk3 (Fig. 4N), embryos with clearly separable Wnt- and Pk3-expressing regions and with apparent membrane localization of Pk3 were selected (*24*).

To evaluate endosomes (Fig. 6H), intracellular vesicles were identified from binarized Wnt11-BFP images using the “Analyze Particles” function in Fiji/ImageJ (see also fig. S10E). Image binarization was performed using the “Triangle” method. Vesicle size was thresholded at 0.04 μm^2^.

To calculate the concentration indices for evaluating signal accumulation (Figs. 4 and 6), the total number of pixels along the cell circumference (Fig. 4) or cell boundary (Fig. 6) was divided by the number of pixels whose intensity exceeded the mean intensity.

### Statistical analyses

Sample sizes were determined empirically. Unless specified in the figure legends, statistical analyses were performed with the Wilcoxon rank-sum test adjusted for multiple comparisons with Holm’s method.
