# Supplementary material for: Planar cell polarity emerges through polarized accumulation of Wnt11
Source: Sci Adv. 2026 Feb 25;12(9):eaea0326. doi: 10.1126/sciadv.aea0326 (PMC12935041; doi:10.1126/sciadv.aea0326)
Supplement: Supplementary file 1 — Figs. S1 to S11 Legend for movie S1 Table S1 [file sciadv.aea0326_sm.pdf]

Science Advances
AAAS

Supplementary Materials for

Planar cell polarity emerges through polarized accumulation of Wnt11

Yusuke Mii *et al.*

Corresponding author: Yusuke Mii, mii.yusuke.7j@kyoto-u.ac.jp; Shinji Takada, stakada@nibb.ac.jp

Sci. Adv. **12**, eaea0326 (2026)
DOI: 10.1126/sciadv.aea0326

The PDF file includes:

Figs. S1 to S11
Legend for movie S1
Table S1

Other Supplementary Material for this manuscript includes the following:

Movie S1

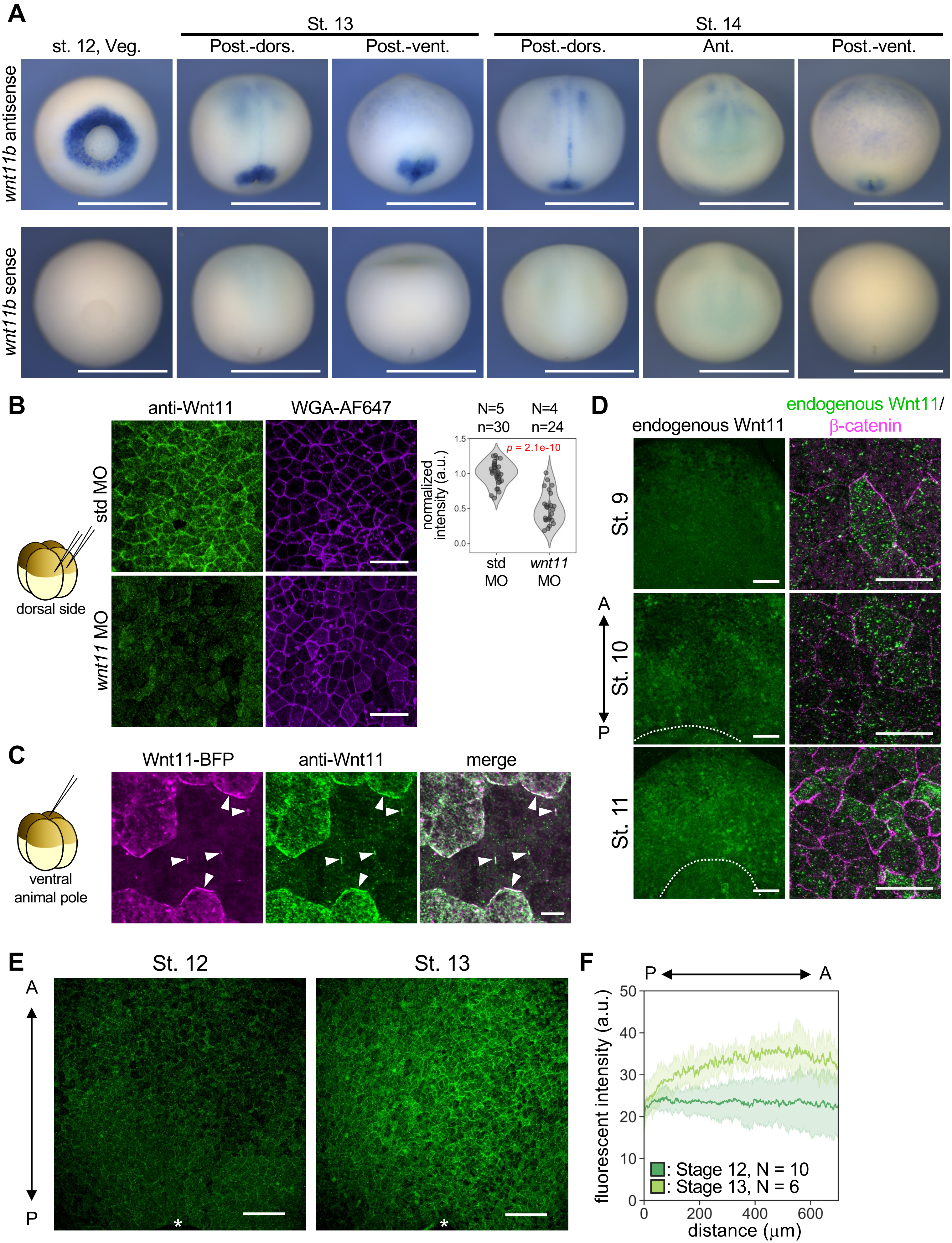
A
St. 13
St. 14
st. 12, Veg.
Post.-dors.
Post.-vent.
Post.-dors.
Ant.
Post.-vent.
wnt11b antisense
wnt11b sense
B
anti-Wnt11
WGA-AF647
std MO
wnt11 MO
dorsal side
N=5
n=30
N=4
n=24
p = 2.1e-10
normalized intensity (a.u.)
1.5
1.0
0.5
0.0
std MO
wnt11 MO
C
Wnt11-BFP
anti-Wnt11
merge
ventral animal pole
D
endogenous Wnt11
endogenous Wnt11/
β-catenin
St. 9
St. 10
St. 11
A
P
E
St. 12
St. 13
A
P
F
P
A
fluorescent intensity (a.u.)
50
40
30
20
10
0
0
200
400
600
distance (μm)
: Stage 12, N = 10
: Stage 13, N = 6

fig. S1. Gene expression pattern of *wnt11* and specificity of anti-Wnt11 immunostaining in *Xenopus* embryos.

A, Gene expression patterns of *wnt11b* visualized by whole-mount *in situ* hybridization. Throughout Stages 12-14, *wnt11b* mRNA is highly localized at the posterior region, near the blastopore. Weaker expression is also observed in the anterior neural plate at Stages 13 and 14.

B, Reduction of anti-Wnt11 staining by knockdown of *wnt11*. Alexa Fluor 647-conjugated wheat germ agglutinin (WGA-AF647) was used as a membrane marker. Numbers of embryos (N) and numbers of subregions (n, squares of 98 μm sides) are as indicated. MOs were injected as illustrated. Amounts of MOs (ng/embryo): *wnt11* MO, 42.7; std MO, 41.6 (both are 5 pmol).

C, anti-Wnt11 staining with overexpression of mTagBFP2-tagged Wnt11 (Wnt11-BFP, W11B). Anti-Wnt11 staining overlapped with localization of Wnt11-BFP, including accumulation and puncta at cell boundaries (arrowheads). *wnt11-BFP* mRNA (1.0 ng/embryo) was injected as illustrated.

D, E, Immunostaining of endogenous Wnt11 at earlier stages. Posterior-dorsal view. The anterior-posterior (AP) axis is as indicated. Dashed lines and asterisks indicate positions of the dorsal edge of blastopore. At Stage 11 or earlier, Wnt11 staining was mostly observed inside cells. After Stage 12, staining at cell boundaries (indicated with β-catenin) become apparent.

F, Spatial profile of Wnt11 in the dorsal/neural plate region at earlier stages. Wnt11 did not form a concentration gradient from posterior to anterior in dorsal ectoderm of St. 12 or in the neural plate of St. 13. Fluorescent intensities were obtained from line plots of 152 μm width at the center of the dorsal/neural plate region. Error ranges are given as standard deviations (s.d.). Numbers of embryos (N) are as indicated.

Scale bars: 1 mm (A), 50 μm (B, D-left column), 10 μm (C), 200 μm (D-right column, E). Representative data from two independent experiments are presented.

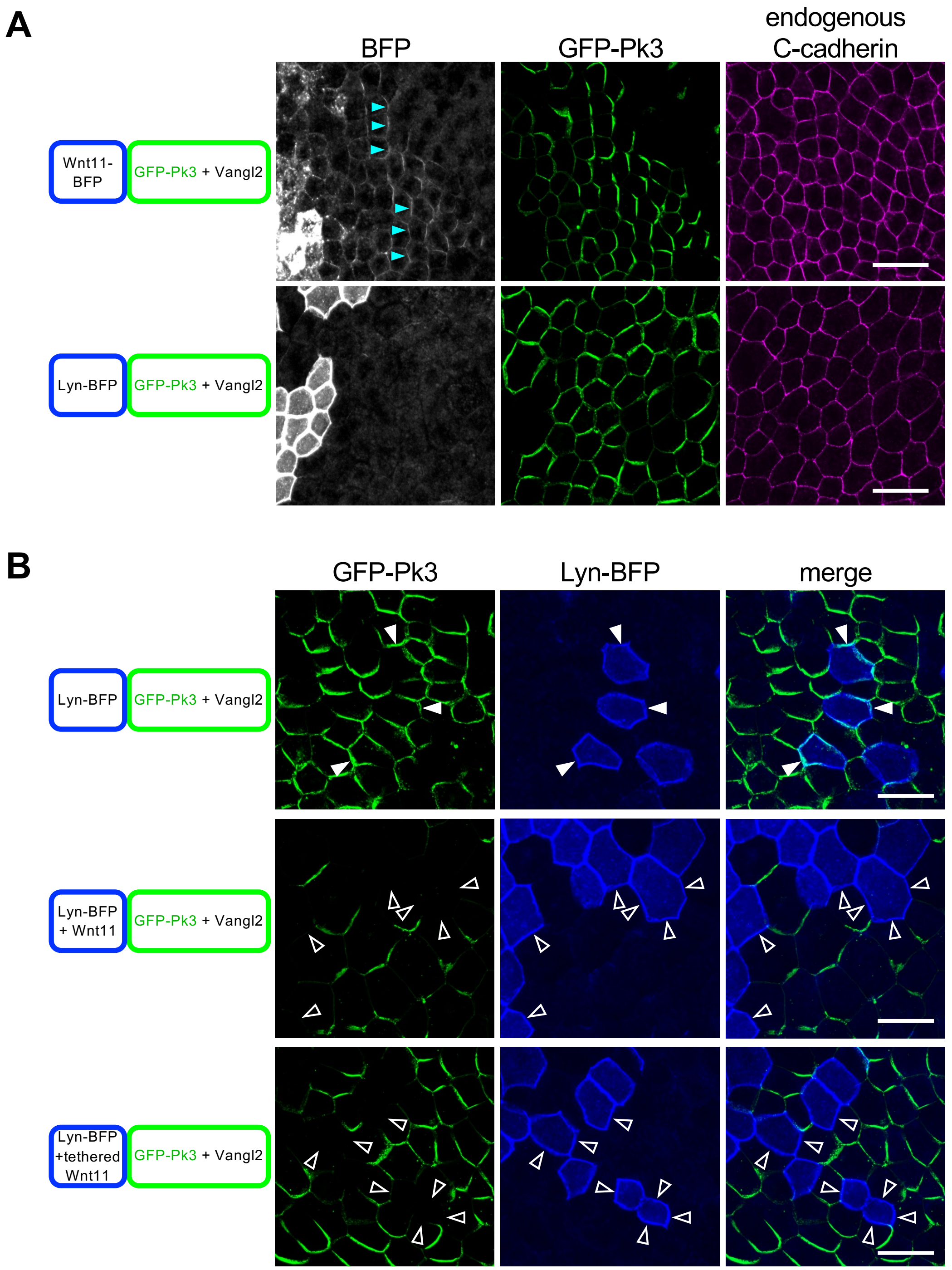
A
BFP
GFP-Pk3
endogenous C-cadherin
Wnt11-BFP
GFP-Pk3 + Vangl2
Lyn-BFP
GFP-Pk3 + Vangl2
B
GFP-Pk3
Lyn-BFP
merge
Lyn-BFP
GFP-Pk3 + Vangl2
Lyn-BFP + Wnt11
GFP-Pk3 + Vangl2
Lyn-BFP +tethered Wnt11
GFP-Pk3 + Vangl2

fig. S2. Activity of BFP-tagged and membrane-tethered Wnt11 in PCP formation.

A, Reconstructed PCP (rPCP) formation with Wnt11-BFP in the animal cap region. Wnt11-BFP can polarize GFP-Pk3 co-expressed with HA-Vangl2 (upper panels), whereas a membrane tracer, Lyn-BFP cannot (bottom panels). As with endogenous Wnt11 in the neural plate, Wnt11-BFP exhibits polarized localization, co-localized with GFP-Pk3 (asterisks). A membrane marker, C-cadherin is uniformly distributed regardless of polarization of GFP-Pk3.

B, Cell membrane localization of GFP-Pk3 was reduced by both wild-type and membrane-tethered Wnt11 expressed in adjacent cells. Membrane localization of GFP-Pk3 was reduced by both wild-type (WT) and membrane-tethered-Wnt11 expressed in adjacent cells (open arrowheads), but not by Lyn-BFP (closed arrowheads). Note that reduction of GFP-Pk3 occurred at cell boundaries in various directions, suggesting contact-dependent effects of Wnt11-source cells.

mRNAs were injected into ventral animal blastomeres at the 32-cell stage as illustrated. Amounts of mRNAs (pg/embryo): *wnt11-BFP*, 500; *lyn-BFP*, 50; *GFP-pk3*, 200; *HA-vangl2*, 100; *wnt11*, 500; *tethered-wnt11*, 500. Scale bars, 50 μm. Representative data from two independent experiments are presented.

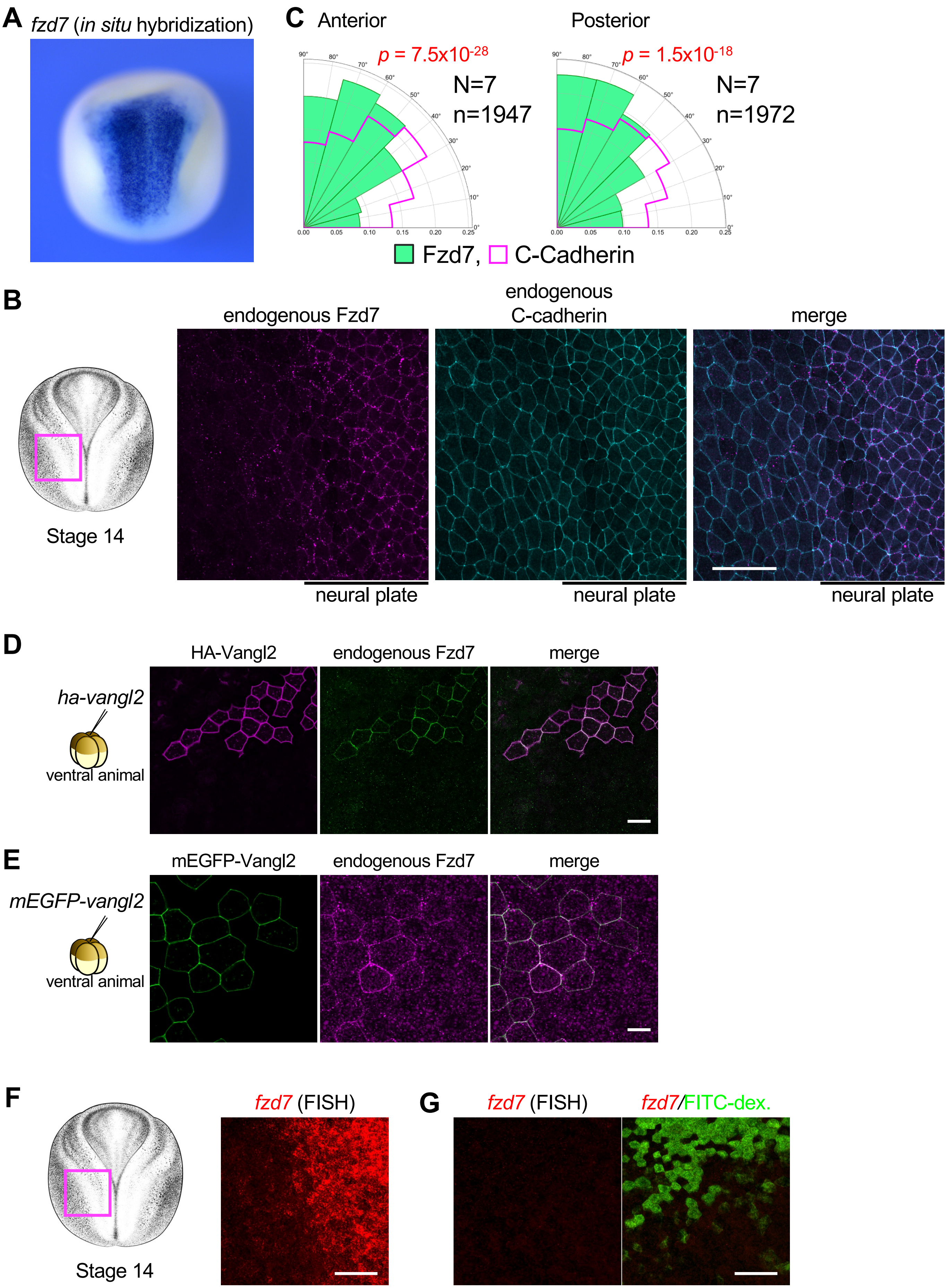
A
fzd7 (in situ hybridization)
C
Anterior
p = 7.5x10-28
N=7
n=1947
Posterior
p = 1.5x10-18
N=7
n=1972
Fzd7, C-Cadherin
B
Stage 14
endogenous Fzd7
endogenous C-cadherin
merge
neural plate
neural plate
neural plate
D
ha-vangl2
ventral animal
HA-Vangl2
endogenous Fzd7
merge
E
mEGFP-vangl2
ventral animal
mEGFP-Vangl2
endogenous Fzd7
merge
F
Stage 14
fzd7 (FISH)
G
fzd7 (FISH)
fzd7/FITC-dex.

fig. S3. Exogenous Vangl2 stabilizes Fzd7 protein on membranes.

A, Gene expression patterns of *fzd7* mRNA visualized by whole-mount *in situ* hybridization. *fzd7* is highly expressed in the neural plate.

B, Membrane localization of endogenous Fzd7 was specifically observed in the neural plate. A boundary region of the neural plate was observed as illustrated. C-cadherin was stained as a membrane marker.

C, Quantification of the polarity angle of endogenous Fzd7 localization in the neural plate. Anterior and posterior halves of neural plates were analyzed. In both regions, Fzd7 was significantly polarized toward 90º (corresponding to localization on the medio-lateral boundaries) compared to C-cadherin.

D, E, Overexpression of Vangl2 in the animal cap region increased membrane localization of Fzd7. HA-Vangl2 (D) or mEGFP-Vangl2 (E) was ectopically expressed. Both increased membrane localization of endogenous Fzd7.

F, G, Fluorescent *in situ* hybridization (FISH) of *fzd7*. (F) Boundary of the neural plate. Similar to normal *in situ* hybridization (A), *fzd7* transcripts were intensively detected in the neural plate. (G) HA-*vangl2* mRNA and FITC-dextran were injected into a ventral animal blastomere of 32-cell stage embryos. Overexpression of HA-Vangl2 did not increase *fzd7* transcripts in the animal cap region, suggesting that the increase of Fzd7 by Vangl2 overexpression is probably due to stabilization of Fzd7 protein, but not due to an increase of *fzd7* transcripts. Samples were stained and visualized in a side-by-side manner to equalize sensitivity for *fzd7* transcripts in F and G.

mRNAs were injected as illustrated. Amounts of mRNAs (pg/embryo): *HA-vangl2*, 50 (D), 100 (G); *mEGFP-vangl2*, 100. Scale bars, 100 μm (B, F, G), 40 μm (D), 20 μm (E). Representative data from two independent experiments are presented. *Xenopus* illustrations © Natalya Zahn (2022) (CC BY-NC, https://creativecommons.org/licenses/by-nc/4.0/deed.en).

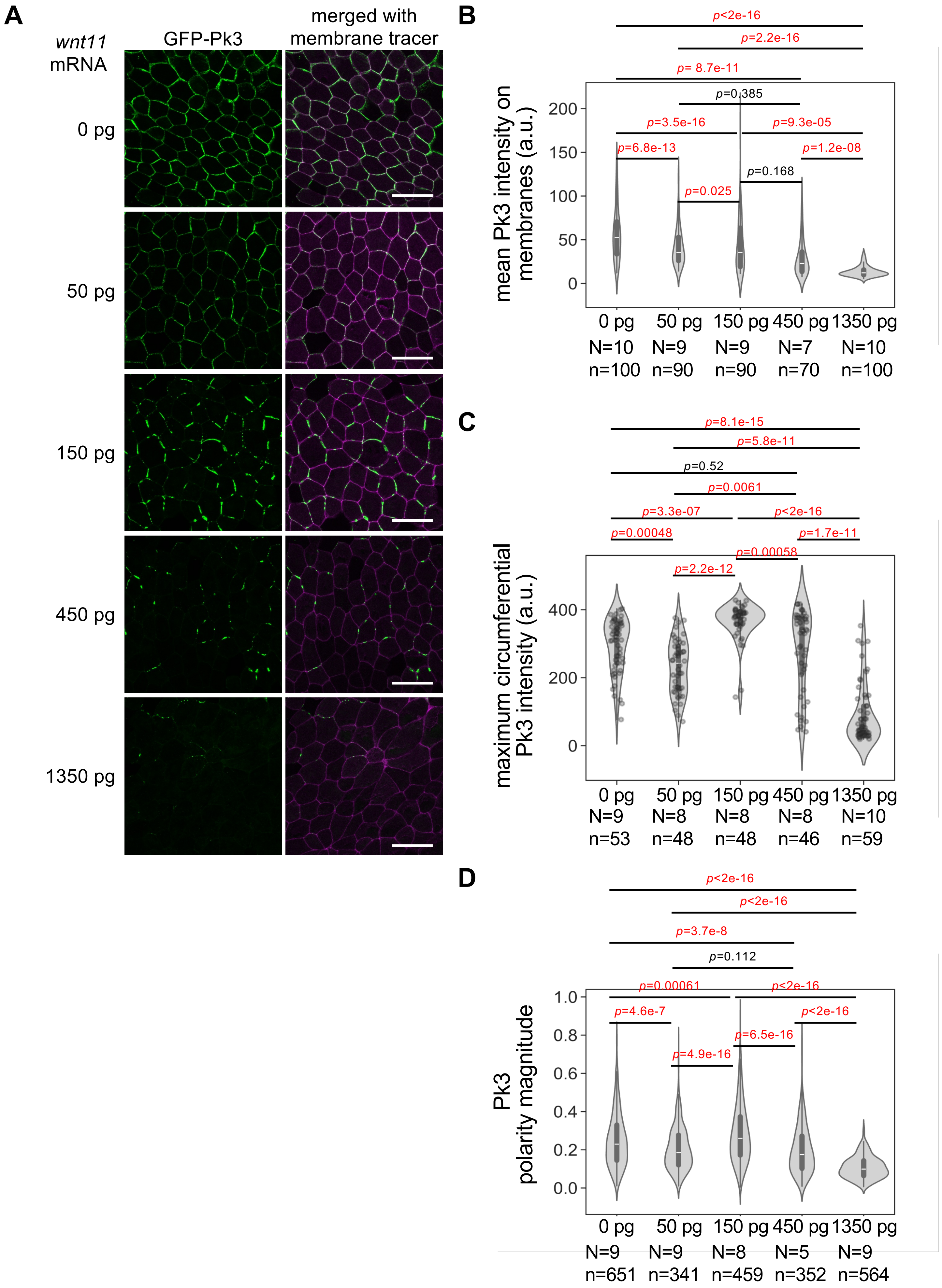
A
wnt11 mRNA
GFP-Pk3
merged with membrane tracer
0 pg
50 pg
150 pg
450 pg
1350 pg
B
mean Pk3 intensity on membranes (a.u.)
p<2e-16
p=2.2e-16
p= 8.7e-11
p=0.385
p=3.5e-16
p=9.3e-05
p=6.8e-13
p=1.2e-08
p=0.168
p=0.025
0 pg 50 pg 150 pg 450 pg 1350 pg
N=10 N=9 N=9 N=7 N=10
n=100 n=90 n=90 n=70 n=100
C
maximum circumferential Pk3 intensity (a.u.)
p=8.1e-15
p=5.8e-11
p=0.52
p=0.0061
p=3.3e-07
p<2e-16
p=0.00048
p=1.7e-11
p=0.00058
p=2.2e-12
0 pg 50 pg 150 pg 450 pg 1350 pg
N=9 N=8 N=8 N=8 N=10
n=53 n=48 n=48 n=46 n=59
D
Pk3 polarity magnitude
p<2e-16
p<2e-16
p=3.7e-8
p=0.112
p=0.00061
p<2e-16
p=4.6e-7
p<2e-16
p=6.5e-16
p=4.9e-16
0 pg 50 pg 150 pg 450 pg 1350 pg
N=9 N=9 N=8 N=5 N=9
n=651 n=341 n=459 n=352 n=564

fig. S4. Dose-dependent reduction and biphasic polarization of GFP-Pk3 by co-expressed Wnt11.

A, Localization of GFP-Pk3.

B, Quantification of mean intensity of GFP-Pk3 on cell membranes. Numbers of embryos (N) and numbers of cell boundaries (n) are as indicated. Wnt11 reduced GFP-Pk3 on membranes in a dose-dependent manner.

C, Maximum circumferential intensities of GFP-Pk3. Unlike the mean intensity, maximum intensity was highest with an intermediate dose of *wnt11* mRNA (150 pg/embryo). Numbers of embryos (N) and numbers of cells (n) are as indicated.

D, Quantification of polarity magnitude of GFP-Pk3. As with the maximum intensity, polarity magnitude was also highest with an intermediate dose of *wnt11* mRNA (150 pg/embryo). Numbers of embryos (N) and numbers of cells (n) are as indicated.

mRNAs were injected into a ventral animal blastomere at the 16-cell stage. mRNAs (pg/embryo): *wnt11*, as indicated; *GFP-pk3*, 200; *HA-vangl2*, 100; *mRuby2-krasCT*, 67. Scale bars, 50 μm. Representative data from two independent experiments are presented.

fig. S5. (Mii et al.)

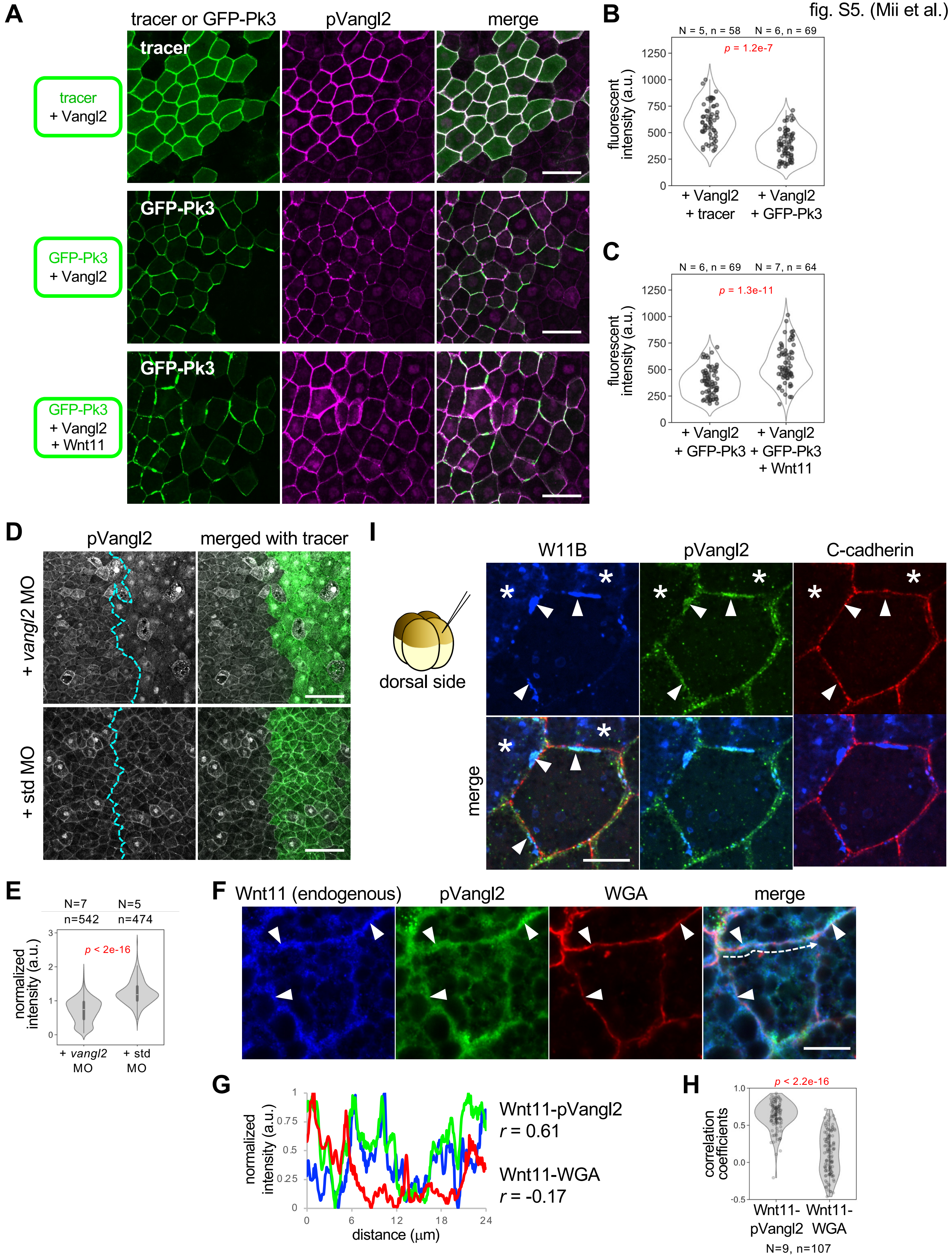

fig. S5. Specificity of anti-phospho Vangl2 (pVangl2) staining.

A-C, pVangl2 staining in overexpression of Vangl2, GFP-Pk3, and Wnt11. (A) mRNAs were injected into the animal pole region of a ventral blastomere at the 4-cell stage. Overexpression of Vangl2 in the animal cap region increased pVangl2 staining, compared to tracer-negative cells. mEGFP-KRasCT was used as a tracer. Co-expression of GFP-Pk3 reduced pVangl2 staining, compared to co-expression of the tracer, consistent with biochemical analyses with *Xenopus* embryos (*50*). Further addition of Wnt11 to co-expression of Vangl2 and GFP-Pk3 increased pVangl2 staining, suggesting that Wnt11 can induce phosphorylation of Vangl2. (B, C) Quantification of staining intensity of pVangl2. A single data point corresponds to an average intensity of a line plot on a cell boundary between tracer/GFP-Pk3-positive and -negative cells. Numbers of embryos (N) and numbers of cell boundaries (n) are as indicated. For (+ Vangl2, + GFP-Pk3), the same data are presented in B and C.

D, E, pVangl2 staining is reduced by *vangl2* MO in the neural plate.

MOs (14 ng/embryo) were injected into a dorsal blastomere at the 4-cell stage, targeting the future neural plate. FITC-dextran was used as a tracer. Intensity of pVangl2 on cell membranes was quantified in injected and uninjected sides, and normalized with the mean value of the uninjected side in each embryo.

F, G, Co-localization of endogenous Wnt11 and pVangl2 at subcellular resolution in the neural plate. Wnt11 and pVangl2 are frequently co-localized in a punctum-by-punctum manner (arrowheads), but not with wheat germ agglutinin (membrane marker).

E, Intensity profiles of Wnt11, pVangl2 and WGA at a cell boundary. Normalized intensities along the dashed arrow (F) are plotted.

H, Quantification of correlation coefficients. Correlation coefficients between Wnt11 and pVangl2 are significantly higher than those between Wnt11 and WGA. 107 cell boundaries from 9 embryos were analyzed. Statistical analysis was performed with the Wilcoxon signed rank test with continuity correction.

I, Overexpression of Wnt11-BFP (W11B) caused co-accumulation of W11B and pVangl2 in the neural plate. Overexpression of W11B in the neural plate often caused self-accumulation at cell boundaries (arrowheads). At these accumulations, W11B co-localized with pVangl2, but not C-cadherin. W11B-expressing cells are indicated with asterisks.

Amounts of mRNAs: *vangl2*, 100; *GFP-pk3*, 200; *mEGFP-krasCT*, 50; *wnt11*, 250; *wnt11-BFP,* 1.0 ng/embryo. Scale bars, 50 μm (A),100 μm (D) 10 μm (I). Numbers of embryos (N) and numbers of cells (n) are as indicated. Representative data from two independent experiments are presented.

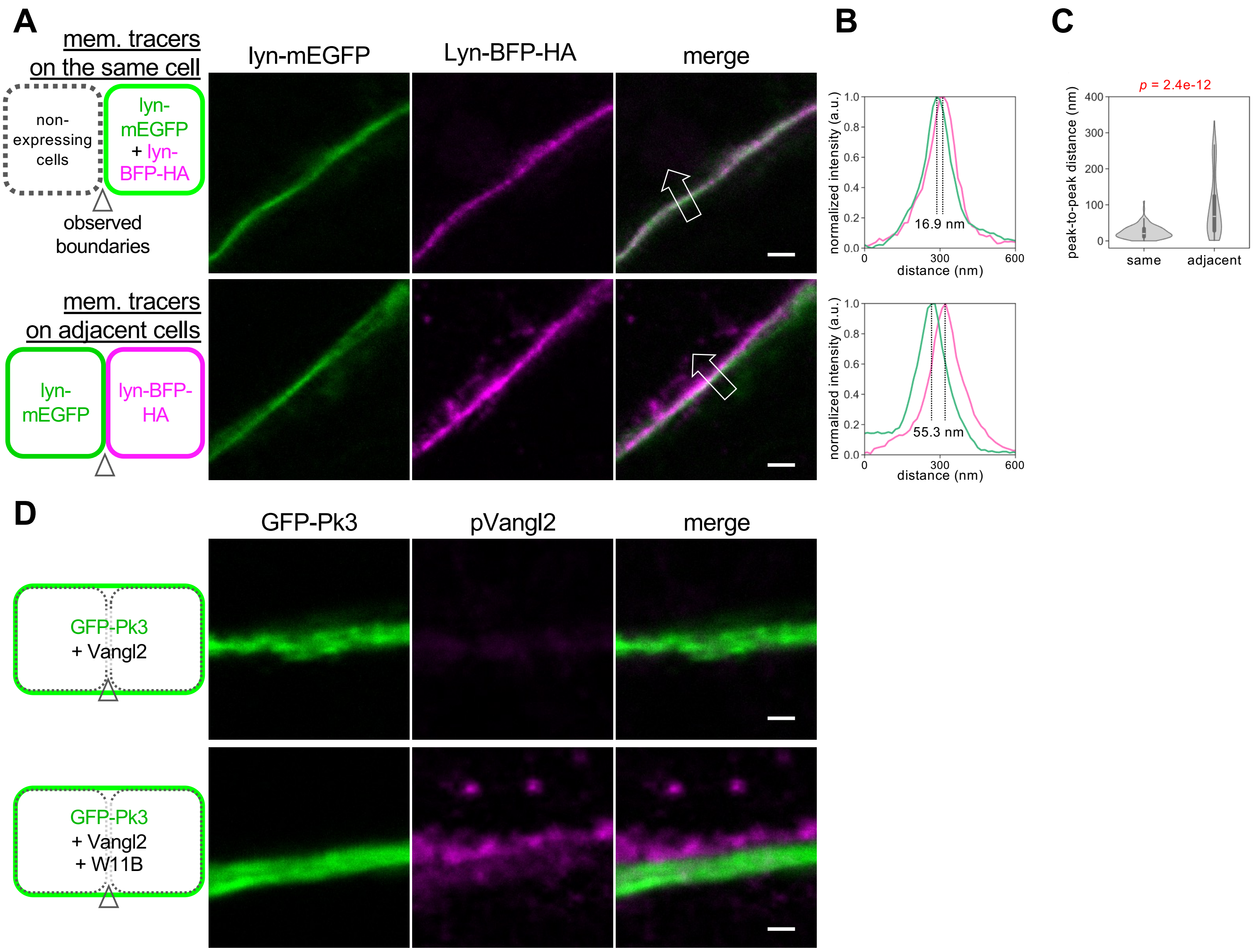
A
mem. tracers
on the same cell
non-
expressing
cells
lyn-
mEGFP
+ lyn-
BFP-HA
observed
boundaries
lyn-mEGFP
Lyn-BFP-HA
merge
mem. tracers
on adjacent cells
lyn-
mEGFP
lyn-BFP-
HA
B
normalized intensity (a.u.)
16.9 nm
distance (nm)
55.3 nm
distance (nm)
C
p = 2.4e-12
peak-to-peak distance (nm)
same
adjacent
D
GFP-Pk3
pVangl2
merge
GFP-Pk3
+ Vangl2
GFP-Pk3
+ Vangl2
+ W11B

fig. S6. Resolution of STED microscopy analyzed by peak-to-peak distance of membrane-tracers.

A-C, STED microscopy can resolve different membrane tracers expressed on adjacent cells. (A) Lyn-mEGFP and Lyn-BFP-3HA were expressed on the same cells or on adjacent cells in the animal cap region of *Xenopus* embryos. (B) Examples of quantification of peak-to-peak distance between the two membrane tracers. Line plots at positions indicated by arrows in A are shown. For a single line, a width of 20 px = 284 nm was measured. (C) Peak-to-peak distance (PPD). A significant difference was observed, indicating that membrane proteins on adjacent cells can be resolved by our STED observations. Data from 4 embryos, 23 cell boundaries, 119 lines (same cells) and 4 embryos, 21 cell boundaries, 115 lines (adjacent cells) from two batches of embryos are presented.

D, pVangl2 staining observed with STED microscopy. In the absence of overexpressed Wnt11-BFP, staining of pVangl2 was weak. In the presence of overexpressed Wnt11-BFP, pVangl2 staining was strong and clearly separated from GFP-Pk3. Representative data from two independent experiments are presented.

mRNAs were injected into animal pole regions of ventral blastomeres at the 4-cell stage, as indicated. Amounts of mRNAs (ng/embryo): *lyn-mEGFP*, 0.10; *lyn-BFP-3HA*, 0.10; *GFP-pk3*, 0.20; *vangl2*, 0.10; *wnt11-BFP*, 1.0. Scale bars, 500 nm.

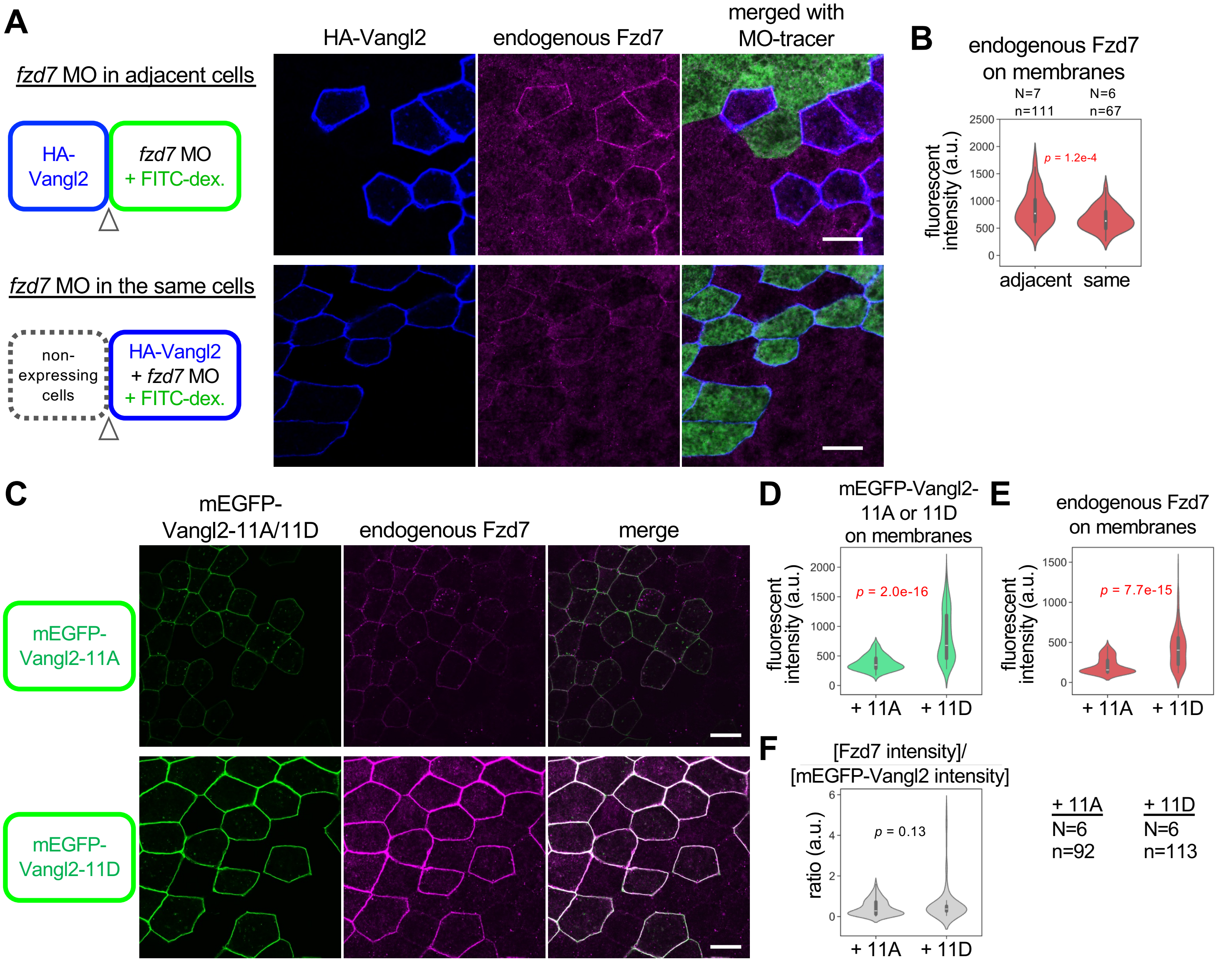
A
HA-Vangl2
endogenous Fzd7
merged with
MO-tracer
fzd7 MO in adjacent cells
HA-
Vangl2
fzd7 MO
+ FITC-dex.
fzd7 MO in the same cells
non-
expressing
cells
HA-Vangl2
+ fzd7 MO
+ FITC-dex.
B
endogenous Fzd7
on membranes
N=7
n=111
N=6
n=67
2500
2000
1500
1000
500
0
p = 1.2e-4
fluorescent
intensity (a.u.)
adjacent
same
C
mEGFP-
Vangl2-11A/11D
endogenous Fzd7
merge
mEGFP-
Vangl2-11A
mEGFP-
Vangl2-11D
D
mEGFP-Vangl2-
11A or 11D
on membranes
2000
1500
1000
500
0
p = 2.0e-16
fluorescent
intensity (a.u.)
+ 11A
+ 11D
E
endogenous Fzd7
on membranes
1500
1000
500
0
p = 7.7e-15
fluorescent
intensity (a.u.)
+ 11A
+ 11D
F
[Fzd7 intensity]/
[mEGFP-Vangl2 intensity]
6
4
2
0
p = 0.13
ratio (a.u.)
+ 11A
+ 11D
+ 11A
N=6
n=92
+ 11D
N=6
n=113

fig. S7. Vangl2 stabilizes Fzd7 on the same membrane in the absence of Wnt11.

A, B, Vangl2 increases membrane localization of Fzd7 on the same membrane. *HA-vangl2* mRNA and *fzd7* MO were injected into ventral animal blastomeres at the 8-16-cell stage as illustrated. (A) Overexpression of HA-Vangl2 increased membrane localization of endogenous Fzd7 (Supplementary Fig. S3). *fzd7* MO in cells adjacent to HA-Vangl2-expressing cells did not affect Fzd7 on membranes (top panels), but *fzd7* MO in the same cells as HA-vangl2 reduced Fzd7 on membranes (bottom panels). (B) Quantification of fluorescent intensity of endogenous Fzd7 on membranes. To confirm which side of Fzd7 was knocked down, cell boundaries between HA-Vangl2-expressing cells and MO-tracer-containing cells, and cell boundaries between HA-Vangl2-expressing cells and non-expressing cells are quantified for "*fzd7* MO in adjacent cells" and "*fzd7* MO in the same cells", respectively. Numbers of embryos (N) and numbers of cell boundaries (n) are as indicated.

C-F, Phospho-deficient and -mimetic mutants of Vangl2 can increase membrane localization of Fzd7 proportionally to the amount of Vangl2 protein on membranes. mEGFP-Vangl2-11A or -11D was overexpressed in the animal cap region. As with wild-type Vangl2, phospho-deficient (11A) and -mimetic (11D) mutants increased endogenous Fzd7 (C). To equalize quantification of proteins on membranes, cell boundaries between mEGFP-Vangl2-11A/D-expressing cells and non-expressing cells are quantified. Amounts of Vangl2 itself (D) and endogenous Fzd7 (E) were significantly higher with 11D-overexpression than those with 11A-overexpression. However, the ratio of [Fzd7 intensity]/[mEGFP-Vangl2 intensity] did not differ significantly between 11A and 11D (F), suggesting that the increase of Fzd7 is proportional to the amount of Vangl2 protein on membranes, regardless of phosphorylation states. Data of 92 cell boundaries from 6 embryos (+ 11A) and 113 cell boundaries from 6 embryos (+ 11D) are presented in D-F.

Amounts of mRNAs/MOs (ng/embryo): *HA-vangl2*, 0.050; *mEGFP-vangl2* (*-11A*, *-11D*); 0.10; *fzd7* MOs, 3.5. Scale bars, 20 μm. Representative data from two independent experiments are presented.

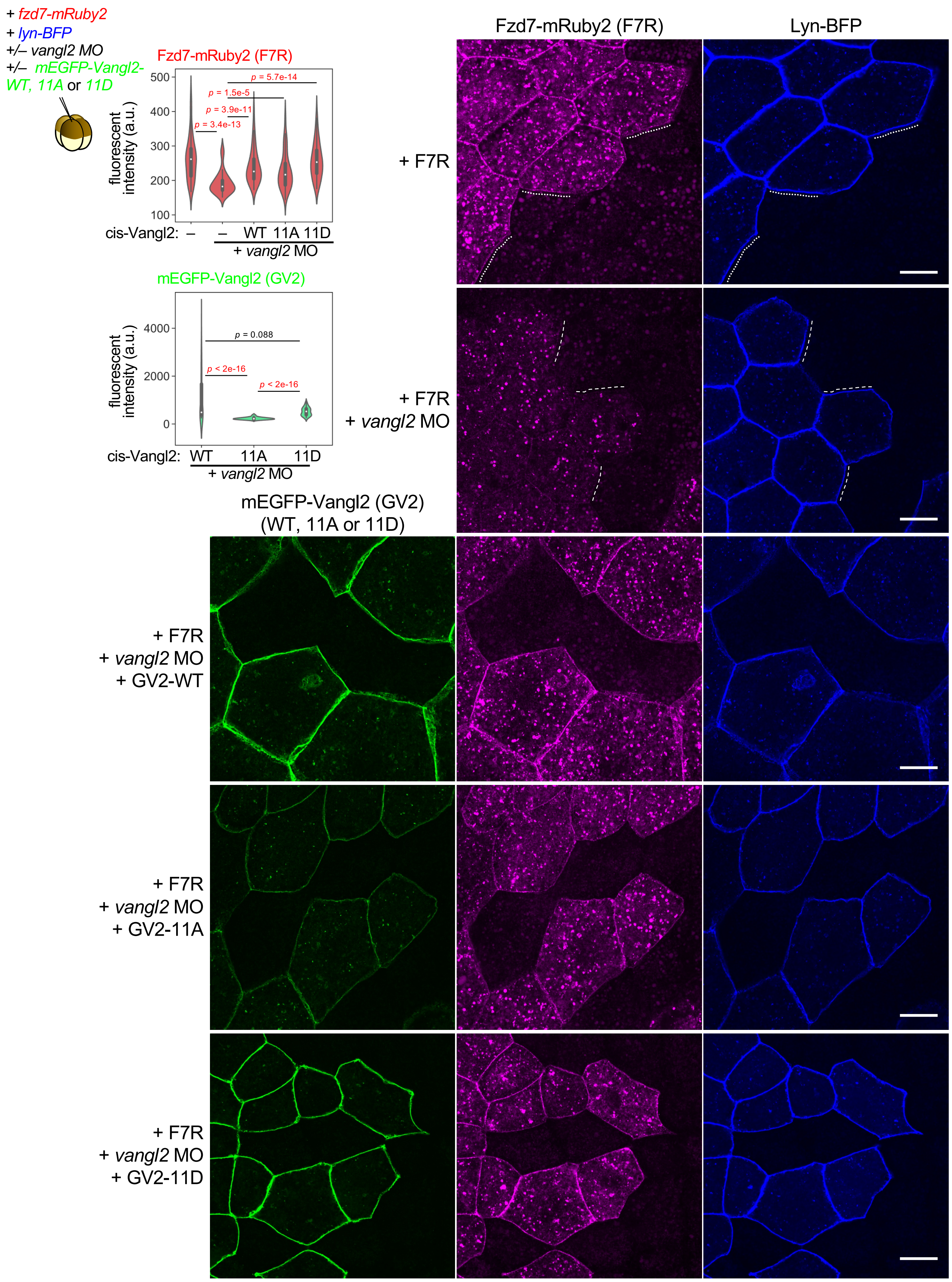

+ fzd7-mRuby2
+ lyn-BFP
+/– vangl2 MO
+/– mEGFP-Vangl2-WT, 11A or 11D
Fzd7-mRuby2 (F7R)
fluorescent intensity (a.u.)
500
400
300
200
100
p = 5.7e-14
p = 1.5e-5
p = 3.9e-11
p = 3.4e-13
cis-Vangl2: – – WT 11A 11D
+ vangl2 MO
mEGFP-Vangl2 (GV2)
4000
2000
0
p = 0.088
p < 2e-16
p < 2e-16
cis-Vangl2: WT 11A 11D
+ vangl2 MO
Fzd7-mRuby2 (F7R)
Lyn-BFP
+ F7R
+ F7R
+ vangl2 MO
mEGFP-Vangl2 (GV2)
(WT, 11A or 11D)
+ F7R
+ vangl2 MO
+ GV2-WT
+ F7R
+ vangl2 MO
+ GV2-11A
+ F7R
+ vangl2 MO
+ GV2-11D

fig. S8. Vangl2 is required for membrane localization of Fzd7 on the same membranes in the absence of Wnt11.

mRNAs and *vangl2* MO were injected into the animal pole of a ventral blastomere as illustrated. (Pictures) Fzd7-mRuby2 (F7R) exhibited membrane localization (dotted lines). Co-injected *vangl2* MO reduced membrane localization of F7R (dashed lines). Additional expression of either the wild-type (WT) mEGFP-Vangl2 (GV2), 11A or 11D can rescue membrane localization of F7R on the same membrane (see cell boundaries between tracer-expressing and non-expressing cells). (Graphs) Cell boundaries between tracer-expressing and non-expressing cells were quantified for amounts of F7R and GV2 on the same membranes. Quantification indicates that GV2-WT and GV2-11D fully rescue membrane localization of F7R, whereas GV2-11A does so partially. In this experiment, translation of F7R is equalized because it completely depends on injected mRNA. Thus, Vangl2 is likely to stabilize Fzd7 on the same membrane, regardless of phosphorylation state. 75 cell boundaries from 5 embryos for each experimental group were quantified.

Amounts of mRNAs/MOs (ng/embryo): *fzd7-mRuby2*, 0.10; *mEGFP-vangl2* (WT, *11A* and *11D*), 0.10; *lyn-BFP*, 0.025; *vangl2* MO, 14. Scale bars, 20 μm. Representative data from two independent experiments are presented.

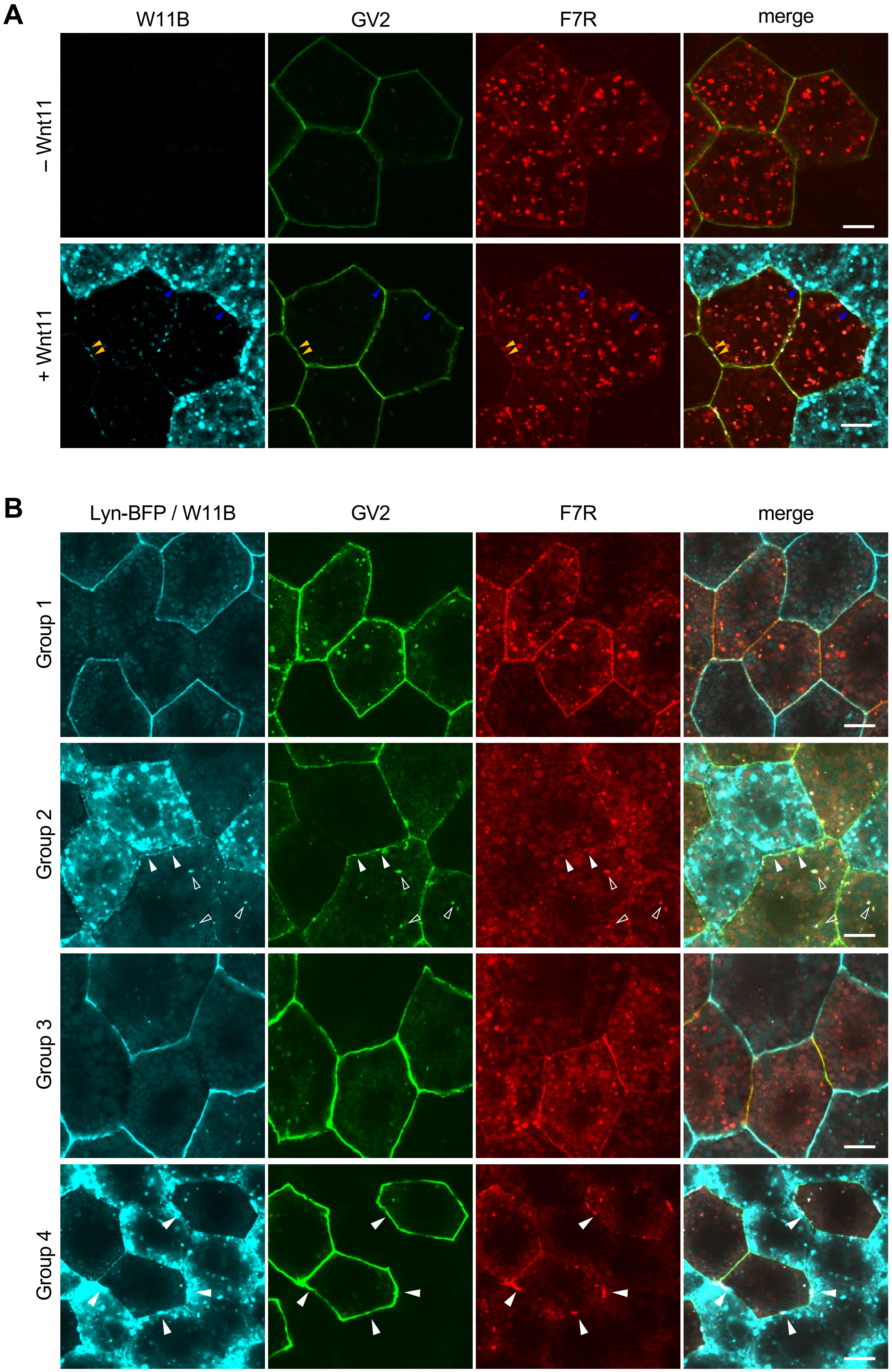
A
W11B
GV2
F7R
merge
− Wnt11
+ Wnt11
B
Lyn-BFP / W11B
GV2
F7R
merge
Group 1
Group 2
Group 3
Group 4

fig. S9. Higher magnification images for Fig. 6A and 6D.

Higher magnification images for Fig. 6A (A) and 6D (B) are presented. Scale bars, 10 μm.

fig. S10. (Mii et al.)

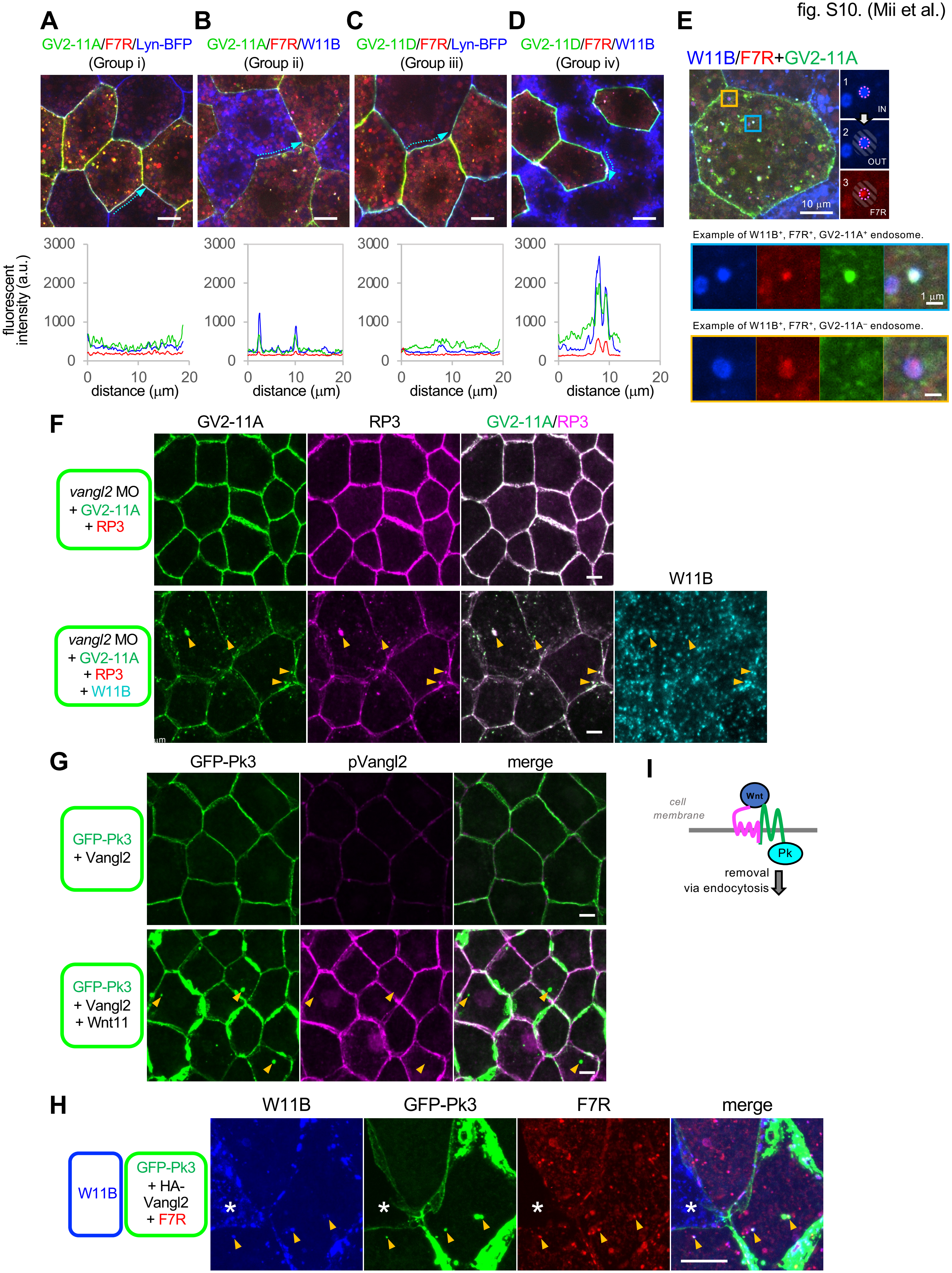

fig. S10. Effects of Wnt11 on Vangl2 and Fzd7 on the same membranes and endosomal removal of Pk3.

A-D, Intensity profiles of each signal along outer cell boundaries shown by dotted arrows (blue, W11B; green, GV2; red, F7R). W11B, GV2 and F7R showed co-accumulation when W11B was co-expressed. Accumulation of these proteins is much higher with GV2-11D than with GV2-11A. Images are the same as Fig. 6D.

E, Analysis of W11B-containing endosomes. W11B-positive puncta inside receiving cells must be internalized from the extracellular space and were identified as endosomes by thresholding the image of W11B. Inside was defined as ROI_{IN} (1). Then, surrounding area was defined as ROI_{OUT} (2). For GV2-11A/D and F7R, presence or absence was determined with mean intensity of ROIs as following (3); $ROI_{IN} > ROI_{OUT}$: positive. $ROI_{IN} < ROI_{OUT}$: negative.

F, Reduction of mRuby2-Pk3 (RP3) from cell membranes and endosomal internalization with GV2-11A by Wnt11. Membrane localization of RP3 was decreased and endosome-like puncta (arrowheads) were increased by co-expression of W11B.

G, Endosome-like puncta of GFP-Pk3 (arrowheads) did not overlap pVangl2 staining.

H, GFP-Pk3 is endocytosed with F7R and W11B. Because W11B is expressed in adjacent cells, W11B-positive puncta inside GFP-Pk3-, HA-Vangl2- and F7R-expressing cells must be internalized by endocytosis.

I, Schematic illustration of endosomal removal of Pk3-non-phosphorylated Vangl2-Fzd7 cis-complexes with Wnt11.

mRNAs were injected into the animal pole region of ventral blastomeres at the 4-cell stage, as illustrated. Amounts of mRNAs/MOs (ng/embryo): *mEGFP-vangl2-11A*, 0.10 (A-E), 0.050 (F); *mEGFP-vangl2-11D*, 0.10; *fzd7-mRuby2*, 0.050 (A-D), 0.10 (E) 0.040 (H); *wnt11*, 0.25; *wnt11-BFP*, 0.50; *lyn-BFP*, 0.050; *GFP-pk3*, 0.20 (G), 0.10 (H); *mRuby2-pk3*, 0.10; *vangl2*, 0.10; *HA-vangl2*, 0.050; *vangl2* MO, 42 (A-D), 21 (F). Scale bars, 10 μm (A-D, E (top panel), F and G), 1 μm (E (middle and bottom panels)). Representative data from two independent experiments are presented.

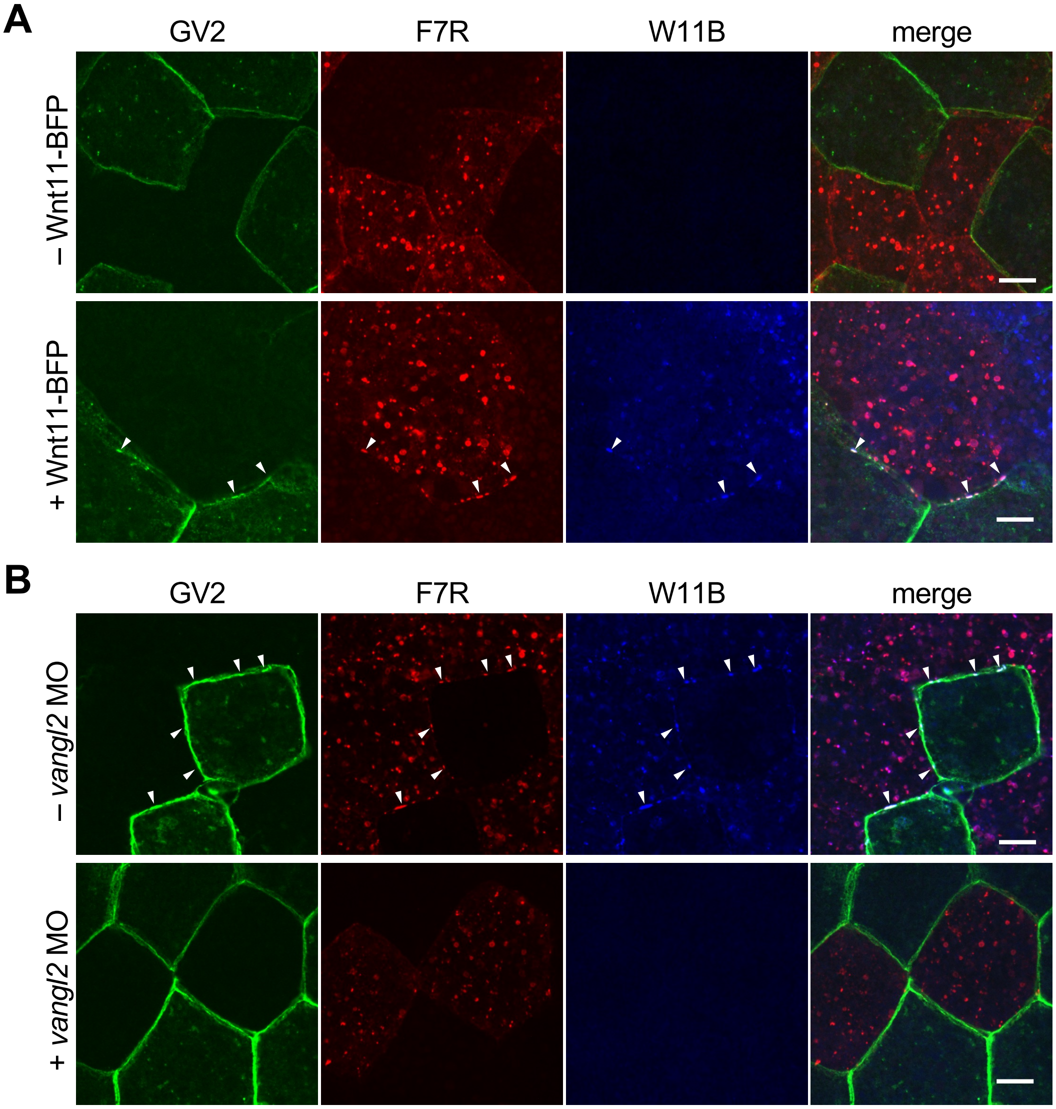
A
GV2
F7R
W11B
merge
− Wnt11-BFP
+ Wnt11-BFP
B
GV2
F7R
W11B
merge
− vangl2 MO
+ vangl2 MO

C

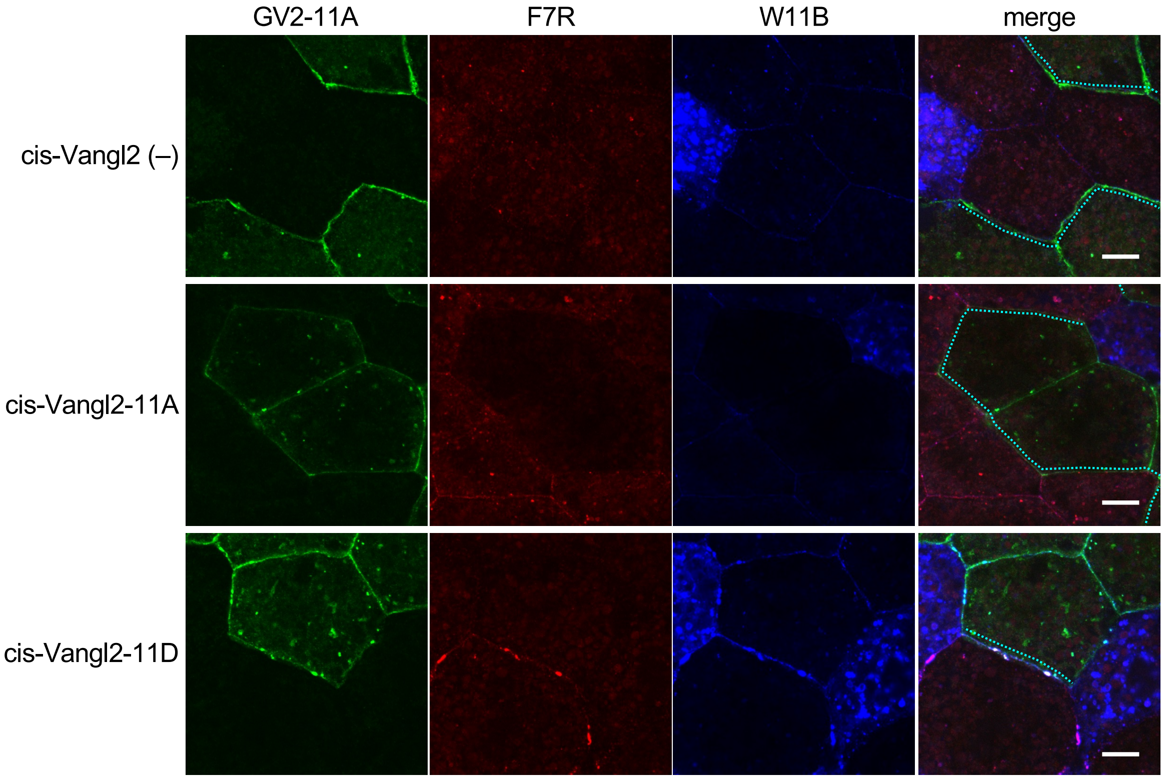
GV2-11A
F7R
W11B
merge
cis-Vangl2 (–)
cis-Vangl2-11A
cis-Vangl2-11D

D

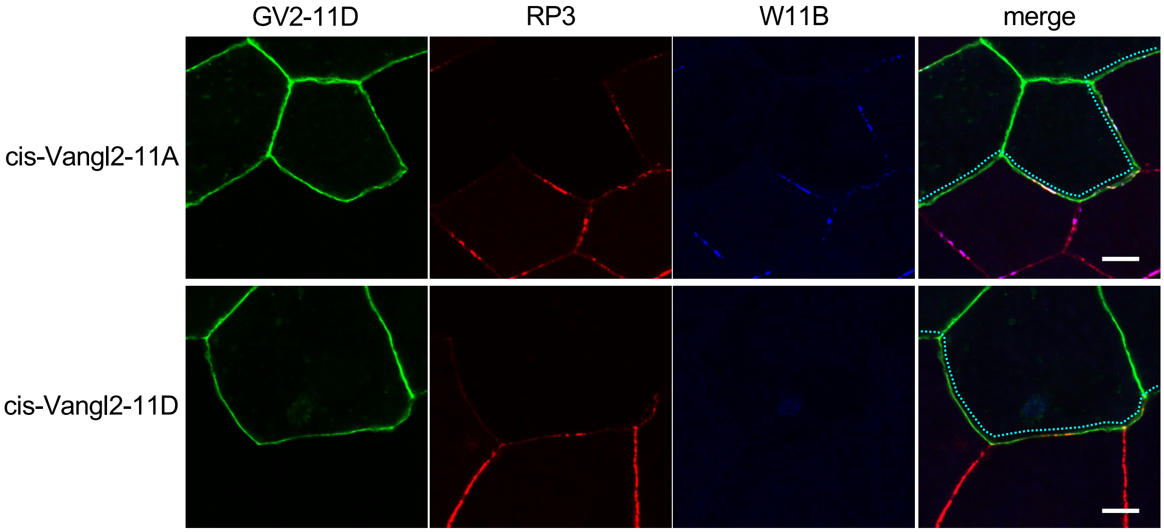
GV2-11D
RP3
W11B
merge
cis-Vangl2-11A
cis-Vangl2-11D

fig. S11. Images of individual channels of trans-assembly of core PCP components by Wnt11.

Images of individual channels corresponding to Fig. 8A (A), 8C (B), 8E (C) and 8G (D). Scale bars, 10 μm.

movie S1. Time-lapse imaging of rPCP formation in the animal cap region.

Live-imaging of GFP-Pk3 and mRFP1 (tracer for Wnt11) during PCP formation (total duration: 9 hours, St. 10.25-14). One second in the movie corresponds to actual one hour. Representative data from three independent experiments are presented.

Table S1

Reagent type or resource	Designation	Source or reference	Identifiers	Additional information	RRID
Antibody	Anti-HA (3F10 rat IgG1)	Roche	Cat no. 11867431001	1:2000	AB_390919
Antibody	Anti-XWnt11 (mouse monoclonal IgG2b)	this study	Clone: #56-1	1:10	
Antibody	Anti-C-Cadherin (mouse monoclonal IgG1)	Developmental Studies Hybridoma Bank	Clone: 6B6	1:50	AB_528113
Antibody	Anti-b-catenin (rabbit polyclonal IgG)	Sigma	Cat no. C2206	1:2000	AB_476831
Antibody	Anti-Vangl2 (rabbit polyclonal IgG)	Sigma	Cat no. HPA027043	1:200	AB_10601709
Antibody	Anti-Phospho-Vangl2-T78/S79/S82	ABclonal	Cat no. AP1206	1:200	
Antibody	Anti-Fzd7 (rabbit polyclonal IgG)	Abcam	Cat no. ab64636	1:200	AB_1640522
Antibody	anti-mouse IgG Alexa Fluor 488 (goat)	Molecular Probes	Cat no. A11001	1:500	AB_2534069
Antibody	anti-mouse IgG Alexa Fluor 647 (goat)	Molecular Probes	Cat no. A21235	1:500	AB_2535804
Antibody	anti-rabbit IgG Alexa Fluor 488 (goat)	Molecular Probes	Cat no. A11008	1:500	AB_143165
Antibody	anti-rabbit IgG Alexa Fluor 555 (goat)	Molecular Probes	Cat no. A21429	1:500	AB_2535850
Antibody	anti-rat IgG Alexa Fluor 555 (goat)	Molecular Probes	Cat no. A21434	1:500	AB_2535855
Lectin	Wheat germ agglutinin (WGA)-conjugated with Alexa Fluor 647	Molecular Probes	Cat no. W32466	1:500	
Software, algorithm	QuantifyPolarity2.0	(49)			
Software, algorithm	Cellpose	(78)			
Software, algorithm	TissueAnalyzer	(79)			
Software, algorithm	MATLAB	Mathworks			
Software, algorithm	ImageJ/Fiji (v1.54f)				
Software, algorithm	RStudio				
Software, algorithm	Spyder				
